# Supplementary material for: An unusual glycerol-3-phosphate dehydrogenase in Sulfolobus acidocaldarius elucidates the diversity of glycerol metabolism across Archaea
Source: Commun Biol. 2025 Apr 1;8:539. doi: 10.1038/s42003-025-07953-9 (PMC11962113; doi:10.1038/s42003-025-07953-9)
Supplement: Supplementary file 1 — Supplementary information [file 42003_2025_7953_MOESM1_ESM.pdf]

Supplementary information

An unusual glycerol-3-phosphate dehydrogenase in *Sulfolobus acidocaldarius* elucidates the diversity of glycerol metabolism across Archaea

Christian Schmerling[1§], Carsten Schroeder[1§], Xiaoxiao Zhou[1§], Jan Bost[2], Bianca Waßmer[2], Sabrina Ninck[3], Tobias Busche[4], Lidia Montero[5,6,7], Farnusch Kaschani[3,8], Oliver J. Schmitz[5,6], Jörn Kalinowski[4], Markus Kaiser[3], Sonja-Verena Albers[2], Christopher Bräsen[1*], Bettina Siebers[1*]

[1]Molecular Enzyme Technology and Biochemistry (MEB), Environmental Microbiology and Biotechnology (EMB), Centre for Water and Environmental Research (CWE), Faculty of Chemistry, University of Duisburg-Essen, Essen, Germany

[2]Molecular Biology of Archaea, Institute of Biology II – Microbiology, University of Freiburg, Freiburg, Germany.

[3]Chemical Biology, Center of Medical Biotechnology, Faculty of Biology, University of Duisburg-Essen, Essen, Germany

[4]Center for Biotechnology (CeBiTec), Bielefeld University, Bielefeld, Germany

[5]Applied Analytical Chemistry (AAC), University of Duisburg-Essen, Essen, Germany

[6]Teaching and Research Center for Separation (TRC), University of Duisburg-Essen, Essen, Germany

[7]Current address: Laboratory of Foodomics, Institute of Food Science Research, CIAL, CSIC, Madrid, Spain

[8]Analytics Core Facility Essen (ACE), Center of Medical Biotechnology, University of Duisburg-Essen, Essen, Germany

§ authors contributed equally

*Correspondence:

Christopher Bräsen - christopher.braesen@uni-due.de

Bettina Siebers – bettina.siebers@uni-due.de

In-depth comparison of structural, enzymatic, and regulatory properties of glycerol kinases across the three domains of life

The glycerol kinase isoenzymes from *S. acidocaldarius*, like all other GKs characterized so far, belong to the FGGY family within the sugar kinase/HSP70/actin superfamily [1]. They exhibit remarkabel sequence conservation, sharing over 50% sequence identity with other archaeal homologues but also with bacterial (>50% sequence identity) and even eukaryotic (~44% sequence identity) enzymes, underscoring their significance across diverse life domains. The homodimeric structure (α_2) of Saci_1117 and Saci_2033 (molecular mass 55.6 kDa and 55.3 kDa, respectively; native molecular weight ~110 kDa, Supplementary Fig. 1 and 2) is consistent with several other GKs found in both eukaryotic (e.g. *Trypanosoma brucei gambiense*, *Plasmodium falciparum*, *Chaetomium thermophilum* [2, 3, 4]) and bacterial (e.g. *Cellulomonas* sp.[5]) organisms. Additionally, various other oligomeric forms including homotetramers (e.g. for some Bacteria like *Thermus thermophilus*, *Elizabethkingia meningoseptica*, and also some Eukarya like *Saccharomyces cerevisiae* [6, 7, 8]) and even homohexamers (*T. kodakarensis* [9]) have been reported.

In case of *E. coli*, in solution a dynamic equilibrium between dimeric and tetrameric states has been documented [10] and also for *Haemophilus influenzae* a higher (>4mer) and a lower (<4mer) oligomerization state was reported [11]. However, Saci_1117 and Saci_2033 showed a consistent homodimeric state in size exclusion chromatography experiments, without evidence of alternative oligomeric forms (Supplementary Fig. 2). Interestingly, the tetramer formation observed in *E. coli* GK is associated with allosteric regulation by fructose 1,6-bisphosphate (F1,6BP) [10], which is absent in both *S. acidocaldarius* GKs, consistent with their homodimeric structure. Similarly, dimeric enzymes from *P. falciparum* and *T. brucei gambiense* also lack allosteric control by F1,6BP [2, 12]. *T. kodakarensis* GK, initially described as a homodimer, did not respond to F1,6BP as an allosteric effector. Subsequent studies revealed that in the presence of glycerol, the *Thermococcus* enzyme assembles into hexamers, although the subunit interactions differ from those observed in *E. coli* tetramers necessary for F1,6BP binding. This finding is consistent with the absence of F1,6BP regulation in *T. kodakarensis* GK [9, 13]. The glycerol-induced hexamer formation in *Thermococcus* GK increases the enzyme's affinity for ATP tenfold. In contrast, under identical glycerol concentrations, Saci_1117 and Saci_2033 GKs retain their homodimeric structure (Supplementary Fig. 2). Notably, the crucial lysine residue at position 271 (K271), which is essential for hexamer formation in *T. kodakarensis* GK, is not conserved in Saci_1117 and Saci_2033.

Furthermore, in *Enterobacteriaceae* (e.g. *E. coli*) and *Firmicutes* (e.g. *Enterococcus, Streptococcus, Bacillus*), two distinct regulatory mechanisms for GK involving components of the phosphoenolpyruvate (PEP)-dependent phosphotransferase system (PTS) for sugar

transport have been elucidated (for literature see [14]). In *E. coli*, the unphosphorylated Enzyme IIA^{Glc} serves as an allosteric inhibitor of GK, while in Firmicutes, GK is phosphorylated by HPr~P at a conserved histidine residue (H232 in *Enterococcus casseliflavus)* located in the loop region where F1,6BP binds (for literature see [14]). This phosphorylation, in the absence of PTS sugars, stimulates the enzyme activity 10 to 15-fold. However, similar regulatory mechanisms involving PTS components are unlikely to occur in *S. acidocaldarius* due to the absence of PTS systems in this organism and Archaea in general, although some exceptions exist in halophiles [15]. Interestingly, in *H. volcanii*, a PTS component protein, a HPr homologue encoded by the ptsH2 gene (HVO_1543), is part of the chromosomal glycerol gene cluster [16, 17]. However, the phosphorylated H232 in the *E. casseliflavus* enzyme is not conserved in the *H. volcanii* GK, nor in Saci_1117 or Saci_2033.

The kinetic constants of the Saci_1117 and Saci_2033 GKs fall within a broad range typical of other GKs (Fig. 4, Table 1, Supplementary Table 3). Both isoenzymes showed GK activity *in vitro*, which followed Michaelis-Menten kinetics. At 50 °C we determined V_{max} values of 45.7 ± 0.25 U mg^{-1} for Saci_1117 and of 88.2 ± 2.5 U mg^{-1} for Saci_2033 (Table 1). At a physiological temperature of 75°C, the V_{max} values for Saci_1117 and Saci_2033 GKs were determined to be 170 ± 9 U mg^{-1} and 340 ± 7.3 U mg^{-1}, respectively, aligning closely with those reported for other GKs (Supplementary Table 3). Both GKs showed less than 2% catalytic efficiency with glyceraldehyde (GA) and dihydroxyacetone (DHA) compared to glycerol (Supplementary Fig. 3a and b), a characteristic also observed for other GKs like the *E. coli* enzyme [18].

The pH and temperature optimum for both enzymes was at 7 (Saci_1117) and 6.5 (Saci_2033) and at 75°C, respectively (Table 1, Supplementary Fig. 3c and d). Furthermore, both GKs were stable against thermal inactivation with half-life times of 45 h and 42 h (Saci_1117) and 12 h and 2 h (Saci_2033) at 70 °C and 80 °C, respectively. However, for Saci_2033 no activity was retained after 1 h at 90°C, while Saci_1117 retained around 40% residual activity after 24 h of incubation (Supplementary Fig. 3e and f). Nevertheless, consistent with its optimal growth temperature of 85°C, the thermal properties of the *T. kodakarensis* GK are more pronounced, with a T_{opt} of 80 °C and a $t_{1/2}$ of 30 min at 100 °C [19, 20]. Similarly, the GK from the thermophilic bacterium *T. thermophilus*, with an optimal growth temperature of around 68°C, exhibits a slightly lower T_{opt} between 50 °C and 70 °C and loses approximately 75% activity after a 30 min incubation at 70 °C [7].

D-glyceric acid, D-glucose, D-sorbitol, D-xylose, xylitol, and *meso*-erythritol were not accepted as substrate by the two *S. acidocaldarius* GKs. Compared to ATP, with GTP and CTP only 10% and 5% (Saci_1117) and 20% and 45% (Saci_2033) residual activity, respectively, were observed (Supplementary Fig. 4a and b). Fructose 1,6-bisphosphate, an inhibitor of the *E. coli* GK [10], had no effect on Saci_1117 and Saci_2033 (Supplementary Fig. 4c and d).

In-depth comparison of structural, enzymatic, and regulatory properties of glycerol-3-phosphate dehydrogenases across the three domains of life

In contrast to the *S. acidocaldarius* GK/G3PDH pathway, the route in *H. volcanii* involves a GlpABC G3PDH, akin to the "anaerobic" pathway found in facultatively anaerobic Bacteria like *E. coli* [17, 21, 22, 23, 24]. In Bacteria and haloarchaea, GlpC and GlpB aid membrane anchoring and electron transfer, respectively, from the catalytic subunit GlpA finally to the (mena)quinone pool in *E. coli* [22, 23]. However, GlpB and C are absent from most Archaea, including GlpD and GlpO homologues. These Archaea, besides GlpK and often GlpF, only possess active GlpA homologues, as demonstrated in *S. acidocaldarius* (this work) and *T. kodakarensis* (Koga et al., 2019).

The two *S. acidocaldarius* G3PDH isoenzymes Saci_1118 and Saci_2032 displayed K_M and V_{max} values of 0.019 ± 0.003 mM (G3P) and 19.7 ± 0.73 U mg^{-1} and of 0.055 ± 0.009 mM (G3P) and 44.5 ± 2.47 U mg^{-1}, respectively, at 70°C, with DCPIP serving as the electron acceptor (Fig. 5b, Table 1). No activity was detected with D-glycerol 1-phosphate (G1P), D-glycerol, D-glyceric acid, D-glyceraldehyde, D-glyceraldehyde 3-phosphate (GAP), and D-phosphoglyceric acid. Additionally, no activity was observed with NAD^+ or $NADP^+$ as electron acceptors, using either G3P or glycerol, DHAP and glycerol 1-phosphate as substrate. Ubiquinone-Q1 was confirmed as the electron acceptor, as evidenced by G3P-dependent decrease of absorption at 280 nm (Fig. 5c). K_M and V_{max} values of 0.086 ± 0.019 mM and 18.66 ± 1.41 U mg^{-1} (for Saci_1118) and 0.179 ± 0.075 mM and 21.1 ± 5.25 U mg^{-1} (for Saci_2032) were determined with ubiquinone-Q1 (Fig. 5d, Table 1). Both enzymes exhibited a pH and temperature optimum of 6.5 and 70°C, respectively (Table 1, Supplementary Fig. 6a and b) as well as a remarkable thermal stability, retaining full activity even after 6 h at 70°C. At 80°C, both G3PDHs had a half-life time of 3 h, and no activity was retained after 1 h of incubation at 90 °C (Supplementary Fig. 6c and d). Non-ionic detergents and phospholipids had no effect on recombinant G3PDH isoenzymes (Supplementary Fig. 7).

Compared to the *S. acidocaldarius* G3PDHs, the TK1393 G3PDH catalytic subunit from *T. kodakarensis* alone exhibited a 4 to 5-fold (Saci_1118) and a 7 to 8-fold (Saci_2032) lower V_{max} of 5 - 6 U mg^{-1} and a 50- to over 100-fold higher K_M value for G3P (2.6 mM vs. 0.019 ± 0.003 mM (Saci_1118) and 0.055 ± 0.009 mM (Saci_2032)). However, in Archaea, G3PDH encoding genes are often co-localized downstream with different, likely functionally related genes (for detailed discussion see below), suggesting potential differences in electron transfer, especially given the absence of GlpB and GlpC subunits. We provide evidence that the *S. acidocaldarius* G3PDHs form catalytically active dimers, capable of directly reducing the (ubi)quinone analogue Q1, similar to characterized bacterial and eukaryotic GlpDs [25, 26, 27], which directly transfer the electrons to the quinone pool. Using quinones ($E^{0'}$ ~+110 mV

(ubiquinone) and -75mV (menaquinone)) as acceptors for the relatively high potential electrons of G3P (around ~-200 mV) oxidation is advantageous, as using $NAD(P)^+$ (with E0' of -320 mV) would render this reaction endergonic [28]. Additionally, the FAD content of one per monomer is more similar to the GlpDs. In contrast, the catalytic heterodimer from *E. coli* (GlpAB) contains only one FAD and one FeS cluster, both likely located in the GlpA subunit. The role of the GlpB subunit in the electron transport is not fully understood. Sequence analyses suggest that the GlpB subunit, homologous to several flavoproteins like the FAD subunit of the succinate dehydrogenase, may contain another flavin cofactor involved in electron transport to the FeS cluster(s) in GlpC [22]. The specific activities of *S. acidocaldarius* G3PDHs with DCPIP as artificial electron acceptor were similar to those reported for GlpD from *Vibrio alginolyticus* with the artificial redox carriers PMS-MTT or ferricyanide [25] (Supplementary Table 4). GlpDs from *E. coli* exhibited roughly ten-fold lower activity with DCPIP, even further reduced in the pig brain mitochondrial enzyme (0.8 U mg^{-1}). However, the pig brain enzyme displayed markedly lower kinetic constants for the native substrates ubiquinones-Q0 and -Q1, with V_{max} 2.6 U mg^{-1} and K_M 0.013 mM for ubiquinone-Q1 [29], compared to *S. acidocaldarius* enzymes (V_{max} 18.7 ± 1.41 U mg^{-1}, K_M 0.086 ± 0.019 mM (Saci_1118); V_{max} 21.1 ± 5.25 U mg^{-1}, K_M 0.18 ± 0.075 mM (Saci_2032)), resulting in a similar catalytic efficiency. The high substrate specificity observed for the *S. acidocaldarius* G3PDHs, not converting D-glycerol 1-phosphate (G1P), D-glycerol, D-glyceric acid, D-glyceraldehyde 3-phosphate (GAP), and D-phosphoglyceric acid, has also been reported for two isoenzymes from *Acidiphilium* sp. [30]. However, the *S. acidocaldarius* G3PDHs are more similar to GlpAs. Among bacterial GlpA-like enzymes, the GlpAB active heterodimer from *E. coli* is the only one kinetically characterized in some detail. The reported V_{max} value for the *E. coli* enzyme (34.4 U mg^{-1}) falls within a similar range as the *S. acidocaldarius* G3PDH homodimers (19.7 ± 0.73 U mg^{-1} (Saci_1118), 44.5 ± 2.47 U mg^{-1} (Saci_2032)), although the K_M for G3P is 15-fold and 6-fold lower for *S. acidocaldarius* G3PDHs (0.019 ± 0.003 mM (Saci_1118) and 0.055 ± 0.009 mM (Saci_2032) vs. 0.34 mM for the *E. coli* GlpAB heterodimer) [31]. Conversely, the direct reduction of quinone analogues was not described for the *E. coli* enzyme, likely because it relies on the GlpC subunit for both membrane anchoring and quinone reduction (electron transfer), underscored by the presence of a FeS cluster(s) in the C subunit [23].

In *S. acidocaldarius*, G3PDH and CoxG homologues, *saci_1118/1119* and *saci_2032/2031*, are organized in operons, with proteins from each operon interacting. CoxG functions to recruit G3PDH to the membrane, anchoring it but not directly facilitating electron transfer. Unlike the homodimeric "aerobic" G3PDH (GlpD) in *E. coli*, which directly interacts with the membrane as a monotopic protein and transfers electrons to the ubiquinone pool [27], *S. acidocaldarius* G3PDHs do not require such membrane interaction for their activity. In *E. coli*, GlpD relies on membrane interaction for conformational stability and function, while this dependency is absent

in *S. acidocaldarius* G3PDHs, indicating different membrane associations and potentially distinct mechanisms of action (Fig. 9).

Extended analysis of sequence and structural comparisons of G3PDHs

The sequence and structural analyses demonstrate homology among *S. acidocaldarius* and other archaeal G3PDHs in their N-terminal region, characterized by the D-amino acid oxidase (DAAO) superfamily fold, and further reveal that these proteins share a 'glutathione-reductase-2' type FAD-binding domain and an antiparallel β-sheet-based substrate-binding domain [32]. Unlike glycine oxidase, which comprises only the DAAO fold without any C-terminal extensions, GlpD, GlpO, GlpA proteins, and their archaeal counterparts exhibit variations in both length and domain organization of their C-terminus (Supplementary Figs. 9, 10, 11, and 12). The C-terminus of GlpO from *Streptococcus* sp. (2rgo) features a 140 aa G3P oxidase domain (pfam 16901). In GlpD from *E. coli* (2qcu) this G3P oxidase domain is truncated, lacking a twenty amino acids comprising helix at its very C-terminal end (Supplementary Figs. 10 and 11a, b). Additionally, GlpOs differ from GlpDs by a ~50 amino acid insertion in the C-terminal part of the DAAO fold (Supplementary Fig. 9). The first part of the C-terminal domain of GlpA shares similarities with the C-terminal domain of GlpOs (and thus also of GlpDs). However, the extension is even more pronounced with an additional 160 amino acids comprising a bfd-like domain with an FeS cluster (pfam04324) and an additional α helix at its C-terminal end [1, 33, 34, 35]. The four cysteine residues essential for FeS cluster binding, highly conserved in the C-terminus of GlpA sequences, are notably absent in GlpDs and GlpOs (Supplementary Fig. 9). The alphafold model of *H. volcanii* GlpA superimposes well with the GlpA model from *E. coli*. However, the halophilic GlpAs have a distinctive C-terminal extension of approximately 30 amino acids. Interestingly, *H. volcanii* GlpC also exhibits an N-terminal extension, which may suggest a structural role in complex formation. Unfortunately, alphafold failed to predict secondary structure or fold for these extensions, making it challenging to identify similar folds and assign functions.

Significantly, there is considerable diversity in the C-terminus of all archaeal G3PDH homologues compared to the GlpO, GlpD, and GlpA enzymes (Supplementary Figs. 9 and 12). For instance, the *Thermofilum pendens* related homologues almost entirely lack a C-terminal extension, with the DAAO fold only minimally extended by a very short α helix of five to six amino acids, if at all (Supplementary Fig. 12a). In *S. acidocaldarius*-like archaeal G3PDH homologues, the C-terminal tail comprises roughly 50 amino acids, forming no known or annotated domain, consisting only of three α helices, and showing no similarities to GlpA, GlpO, or GlpD (Supplementary Fig. 12b). The Thermococcales G3PDHs appear to contain the bfd-fold (pfam04324) similar to GlpA, with four cysteine residues present, although the overall fold of the C-terminal domain appears to be different and somewhat shorter (120 amino acids) (Supplementary Figs. 9 and 12c, d). Additional phylogenetic and structural investigations,

along with gene synteny analyses, indicate that the structural differences observed in the C-terminal domains of GlpDs, GlpOs, and GlpAs are likely associated with their distinct functions.

Expanded Analysis of the Distribution of GK and G3PDH Paralogues

The presence of two GK/G3PDH couples is rare in both Archaea and Bacteria. Instead, it is more common to find two G3PDHs but only one GK. For example, *E. coli* and other facultatively anaerobic bacteria have two different G3PDHs, like GlpABC and GlpD [24, 27, 36, 37, 38, 39], while some organisms like *H. volcanii* have two GlpABC isoenzymes [17, 40]. Two GK paralogues are even rarer in prokaryotes [40, 41, 42]. In cases like *S. acidocaldarius* and *L. rhamnosus*, only one of two GK paralogues is essential and up-regulated, indicating functional specialization. Similarly, in *H. volcanii*, only one of the two G3PDH isoenzymes (GlpABC) is essential and up-regulated [17, 41]. These essential paralogues co-occur with other glycerol metabolism related genes, suggesting functional coherence. In *E. coli*, the two G3PDHs, GlpD and GlpABC, are known to be primarily expressed under different environmental conditions [22, 27, 36]. Similarly, in *P. aeruginosa* the two GK paralogues belong to different regulons, with the non-essential one exhibiting significantly lower GK activity, suggesting a potential role in glycerol breakdown under specific conditions or a different metabolic function altogether [42].

Supplementary Table 1 Growth rate and growth yield on varying concentrations of glycerol and D-xylose.

Carbon source	Growth rate [h^{-1}]	Cell dry weight (CDW) [$g\ L^{-1}$]	Molar growth yield [$g\ CDW\ mol^{-1}$]	Growth yield [$g\ CDW\ g^{-1}$]
**10 mM glycerol**	0,0294 ± 0.0002	0.53 ± 0.03	53.3 ± 3	0.578 ± 0.033
**20 mM glycerol**	0.0280 ± 0.0005	0.85 ± 0.02	42.3 ± 1	0.459 ± 0.011
**40 mM glycerol**	0.0284 ± 0.0006	1.39 ± 0.1	34.7 ± 2.5	0.376 ± 0.028
**0.2% (w/v) D-xylose**	0.0195 ± 0.0005	0.27 ± 0.01	20 ± 0.7	0.133 ± 0.005

Means of n≥3 biological replicates are shown ± standard deviation.

Supplementary Table 2 Differentially expressed genes and proteins given as log2-fold-changes in *S. acidocaldarius* MW00G after growth on glycerol and 0.1% (w/v) N-Z-Amine (NZA) (as control) compared to D-xylose (0.2% (w/v)) identified via RNASeq (transcriptome) and label free quantification (LFQ) (proteome) analyses. For growth on NZA only log2-fold changes higher than 2 are given.

		Transcriptome (log2-fold changes)				Proteome (log2-fold changes)			
	Glycerol concentration	10 mM	20 mM	40 mM	NZA	10 mM	20 mM	40 mM	NZA
**Locus tag**	**Function**								
**Saci_0451**	ESCRT-III	3.75	4.08	3.27	4.93	6.86	7.15	6.32	8.92
**Saci_0942**	CopG family transcriptional regulator	2.10	2.61	2.19	2.82	3.51	3.72	3.68	3.34
**Saci_1050**	ATPase, ParA family	2.96	3.00	2.85	-	2.61	2.56	2.71	-
**Saci_1052**	GYD domain-containing secretory protein	3.87	3.82	3.81	-	5.32	5.38	5.20	-
**Saci_1054**	3-(methylthio)propionyl-CoA ligase	3.39	2.44	2.20	-	4.63	4.52	4.68	-
**Saci_1058**	Xylulokinase	3.12	2.99	2.94	-	2.62	2.58	2.71	-
**Saci_1099**	Betaine-aldehyde dehydrogenase	3.00	2.32	2.17	-	5.40	5.58	5.50	-
**Saci_1228**	Nucleoside triphosphate hydrolase	3.37	3.10	2.56	5.06	3.84	4.00	3.39	5.47
**Saci_1372**	Vesicle-fusing ATPase	3.04	2.25	2.00	4.76	4.05	3.83	4.02	6.80
**Saci_1416**	ESCRT-III	3.91	3.99	3.25	5.60	4.42	4.45	3.85	6.61
**Saci_1616**	Antitoxin (DNA-binding domain)	2.08	2.54	2.76	-	3.29	3.17	3.20	3.01
**Saci_1762**	ABC-type	3.33	3.11	3.24	3.03	3.17	3.05	2.98	2.42
**Saci_1763**	ABC-type	3.54	2.91	3.15	-	3.75	3.82	3.36	3.01
**Saci_1764**	ABC-type	3.53	3.12	3.49	3.41	2.87	2.94	2.89	2.26
**Saci_1765**	ABC-type	3.39	3.17	3.38	3.34	2.89	2.98	2.89	2.24
**Saci_1804**	DUF2173 domain-containing protein	2.39	2.19	2.74	-	4.85	4.94	5.04	-
**Saci_1810**	Energy-coupling factor cobalt transporter, ATP-binding protein	2.83	2.37	2.35	-	3.51	3.53	3.58	-
**Saci_1855**	DUF973 domain-containing membrane protein	7.66	6.92	6.98	-	5.81	5.90	5.88	3.13
**Saci_1856**	DUF973 domain-containing membrane protein	3.74	3.88	3.90	-	4.13	4.15	4.07	-
**Saci_2032**	Glycerol 3-phosphate dehydrogenase	3.92	3.31	2.97	2.90	3.01	2.98	3.10	-
**Saci_2033**	Glycerol kinase	3.96	3.51	3.19	2.37	2.47	2.47	2.24	-
**Saci_2034**	Glycerol uptake facilitator	3.85	3.52	3.20	2.22	4.84	3.59	4.66	-
**Saci_2139**	CBS domain containing protein	4.62	5.54	5.81	2.41	3.51	3.38	3.05	-
**Saci_2188**	Ribonucleoside-diphosphate reductase, subunit beta	3.42	2.99	3.07	-	2.93	2.61	2.36	-
**Saci_2206**	Lipoate-protein ligase A	4.77	4.16	4.13	-	3.68	3.69	3.59	-
**Saci_2208**	3-hydroxyacyl-CoA dehydrogenase	5.30	4.72	4.56	-	9.34	9.38	9.45	-
**Saci_2209**	Acetyl-CoA C-acetyltransferase	4.26	3.63	3.45	-	2.75	2.76	2.54	-
**Saci_2210**	DUF35 OB-fold domain containing protein	5.15	4.12	4.16	-	6.93	6.76	7.02	-
**Saci_2211**	3-(methylthio)propionyl-CoA ligase	2.79	2.58	2.54	-	3.00	2.98	2.87	-
**Saci_2212**	Ribonucleoside-diphosphate reductase, subunit beta	6.32	5.23	5.38	-	3.91	3.95	3.37	-
**Saci_2230**	DUF35 OB-fold domain containing protein	2.94	2.42	2.68	2.94	2.80	2.37	2.74	-

**Saci_2231**	DUF35 OB-fold domain containing protein	3.29	2.63	2.93	3.31	2.95	2.85	2.96	-
**Saci_2232**	Acetyl-CoA C-acetyltransferase	3.39	2.62	2.86	3.31	2.77	2.65	2.61	-
**Saci_2233**	Acetyl-CoA C-acetyltransferase	3.24	2.81	3.07	3.08	2.80	2.73	2.71	-
**Saci_2235**	3-hydroxypropionyl-CoA synthase	5.67	5.07	5.30	-	8.19	8.39	7.99	-
**Saci_2258**	Membrane protein, terminal oxidase function	2.25	2.60	2.84	-	2.48	2.43	2.39	-
**Saci_2259**	Cytochrome B558 subunit B	2.61	2.71	3.02	-	4.61	4.10	4.54	-
**Saci_2261**	Rieske Fe-S protein	2.49	3.23	3.35	-	5.08	4.87	5.16	-
**Saci_2263**	Quinol oxidase subunit 1/3	2.97	3.05	3.13	-	5.85	5.46	5.73	-
**Locus tag**	**Function**								
**Saci_0137**	Phosphomethylpyrimidine synthase	-4.55	-4.23	-4.57	-	-2.86	-2.93	-2.80	-
**Saci_0314**	Hypothetical protein	-5.43	-4.33	-4.15	-4.49	-2.36	-3.06	-2.10	-
**Saci_0526**	Thiazole/oxazole-forming peptide maturase	-2.23	-2.32	-2.03	-	-3.01	-3.33	-3.20	-3.43
**Saci_0531**	ABC-type Mn/Zn transport system	-3.54	-2.59	-2.54	-	-3.76	-3.33	-3.39	-2.89
**Saci_1700**	2,5-dioxovalerate dehydrogenase	-6.09	-5.54	-6.06	-6.07	-7.18	-7.32	-7.08	-6.61
**Saci_1707**	ABC-type molybdate transport system	-5.53	-5.93	-5.31	-5.58	-4.34	-4.79	-5.08	-4.69
**Saci_1830**	Alpha/beta hydrolase	-4.95	-4.19	-4.07	-2.14	-4.16	-4.49	-3.60	-2.28
**Saci_1831**	Transciptional regulator	-4.13	-3.81	-3.81	-	-2.34	-2.04	-2.21	-
**Saci_1938**	2,5-dioxovalerate dehydrogenase	-4.08	-4.01	-4.17	-3.63	-6.83	-6.95	-6.73	-6.70
**Saci_1939**	2-dehydro-3-deoxy-D-arabinonate dehydratase	-3.89	-3.44	-3.77	-3.65	-5.70	-5.50	-5.48	-5.43
**Saci_2090**	Rieske Fe-S protein	-3.12	-2.86	-2.62	-	-3.33	-3.93	-3.91	-
**Saci_2122**	Xylose/arabinose ABC-type transport system	-2.35	-2.43	-2.41	-3.49	-4.07	-4.10	-4.24	-6.56
**Saci_2203**	Sulfate adenylyltransferase	-3.61	-4.16	-4.27	-	-2.16	-2.49	-2.75	-8.21
**Saci_2204**	Hypothetical protein	-3.73	-4.08	-4.25	-7.77	-2.89	-3.60	-3.46	-6.39

Supplementary Table 3 Kinetic parameters, oligomeric state, and (co)substrate spectrum of different characterized GKs selected from all three domains of life.

Enzyme	Organism	Cofactors	Oligomeric state	Substrate	V_{max} [U mg^{-1}]	K_M (glycerol) [mM]	K_M (ATP) [mM]	Literature
**Saci_1117**	*S. acidocaldarius*	Mg^{2+}	homodimer	Glycerol, ATP	170 ± 9 (at 75°C)	0.024 ± 0.001	0.205 ± 0.024	This work
**Saci_2033**	*S. acidocaldarius*	Mg^{2+}	homodimer	Glycerol, ATP	339.7 ± 7.3 (at 75°C)	0.024 ± 0.004	0.174 ± 0.019	This work
**GlpK**	*E. coli*	Mg^{2+}	homodimer/homotetramer	Glycerol, ATP	18.1-100 (at 25°C)	0.0013	0.0078	[10, 18]
**GlpK**	*Thermus thermophilus*	Mg^{2+}	homotetramer	Glycerol, ATP	56.7 (at 37°C)	0.038	0.162	[7]
**GlpK**	*Trypanosoma brucei*	Mg^{2+}	homodimer	Glycerol, ATP	226 (at 25°C)	0.44	0.24	[12, 43]
**GlpK**	*Thermococcus kodakarensis*	Co^{2+}	homodimer/homohexamer	Glycerol, ATP	1500 (at 80°C)	0.111	0.059	[9, 19]

Parameter values obtained in this work are given ± standard deviation.

Supplementary Table 4 Kinetic parameters, oligomeric state, and cosubstrate spectrum of different characterized G3PDHs from selected organisms from all three domains of life. DCPIP: 2,6-Dichlorphenolindophenol; FAD: flavin adenine dinucleotide; G3P: Glycerol-3-phosphate; MTT: (4,5-Dimethylthiazol-2-yl)-2,5-diphenyltetrazoliumbromid; PMS: Phenazine methosulfate; UQ: Ubiquinone.

Enzyme	Organism	Cofactors per monomer	Oligomeric state	Substrate	V_{max} [U/mg]	K_M (for G3P) [mM]	K_M (electron acceptor) [mM]	Literature
**Saci_1118**	*S. acidocaldarius*	1 FAD	homodimer	G3P	19.7 ± 0.73 (DCPIP)<br>18.66 ± 1.41 (UQ -1)	0.019 ± 0.003 (DCPIP)	0.086 ± 0.019 (UQ -1)	This work
**Saci_2032**	*S. acidocaldarius*	1 FAD	homodimer	G3P	44.5 ± 2.47 (DCPIP)<br>21.1 ± 5.25 (UQ -1)	0.055 ± 0.009 (DCPIP)	0.179 ± 0.075 (UQ -1)	This work
**GlpAB**	*E. coli*	1 FAD<br>2 non-heme iron	heterodimer	G3P	34.4 (PMS-MTT)	0.339 (PMS-MTT)		[31]
**GlpA**	*Thermococcus kodakarensis* KOD1	FAD	monomer	G3P	5.63 (MTT)	2.68 (MTT)		[44]
**GlpD**	*E. coli*	2 FAD	homodimer	G3P	5.8 (DCPIP)	0.8 (DCPIP)		[27, 37, 45]
**GlpD**	Pig brain mitochondria	FAD	homodimer	G3P	2.63 (UQ-1<br>0.8 - 2.9 (DCPIP)	6.2 (U-Q10)<br>10 (DCPIP)<br>10 (UQ-0/1/2/6)	0.013 (UQ-1)<br>0.076 (DCPIP)	[29, 46]
**GlpD**	*Vibrio alginolyticus*	FAD	unknown	G3P	39.2 (PMS)<br>85.8 (ferricyanide)	4.02 (PMS)<br>3.7 (ferricyanide)	0.13 (PMS)<br>0.55 (ferricyanide)	[25]
**GlpD**	*Acidiphilium* sp. 63	0.58 FAD	homodimer	G3P	32 (PMS-MTT)	-	-	[30]

Parameter values obtained in this work are given ± standard deviation.

Supplementary Table 5 Identification of *in vivo* interacting proteins with CoxG homologues in *S. acidocaldarius*. HA-tagged CoxG proteins, HA-*saci_2031* and HA-*saci_1119* were expressed in *S. acidocaldarius* and interacting proteins were enriched using co-immunoprecipitation with anti-HA antibodies coupled to magnetic beads. The respective enrichment is given as log2-fold changes.

**Majority protein ID (HA-Saci_1119)**	Gene name ID	Log2-fold changes
**Q4J9R0**	*saci_1119*	14.72
**Q4J9A5**	*saci_1283*	6.76
**Q4JBM7**	*saci_0386*	3.71
**Q4J9Q8**	*saci_1118*	3.45
**Q4J953**	*saci_1337*	3.37
**Q4JB63**	*saci_0574*	2.79
**Q4JAK6**	*saci_0803*	2.21
**Majority protein ID (HA-Saci_2031)**	Gene name ID	Log2-fold changes
**Q4J7A4**	*saci_2031*	11.06
**Q4J7Q9**	*saci_1865*	6.00
**Q4JBM7**	*saci_0386*	4.19
**Q4JB23**	*saci_0614*	2.91
**Q4JAW2**	*saci_0687*	2.83
**Q4JAP0**	*saci_0766*	2.57
**Q4J7A2**	*saci_2032*	2.43
**P13123**	*saci_0064*	2.42
**Q4J6G8**	*saci_2329*	2.15
**Q4J9Q8**	*saci_1118*	2.13

Supplementary Table 6 Strains and Plasmids used in this study.

Designation	Genotype or description	Reference/Source
**Strains**		
MW001	*Sulfolobus acidocaldarius* strain; For growth or deletion mutant construction	[47]
DH5α	*Escherichia coli* strain (DSMZ 6897); Plasmid construction	Hanahan, USA
Rosetta (DE3)	*Escherichia coli* strain; Gene expression; pRARE (chloramphenicol resistance)	Stratagene, USA
MW00G	*Sulfolobus acidocaldarius* strain; glycerol adapted strain	This study
MW1257	*S. acidocaldarius* MW00G Δ*saci_1117*	This study
MW1258	*S. acidocaldarius* MW00G Δ*saci_2033*	This study
MW1259	*S. acidocaldarius* MW00G Δ***saci_1117*** Δ***saci_2033***	This study
MW1257 Δ*saci_1494:P*$_{saci\_1117}$ *saci_1117 CtSS*	*S. acidocaldarius* MW00G Δ*saci_1117* Δ***saci_1494****:P*$_{saci\_1117}$ *saci_1117 CtSS*	This study
MW1258 Δ*saci_1494:P*$_{saci\_2033}$ *saci_2033-2034*	*S. acidocaldarius* MW00G Δ*saci_2033* Δ***saci_1494****:P*$_{saci\_2033}$ *saci_2033-2034*	This study
**Plasmids**		
pET15b	*E. coli* expression plasmid carrying an N-terminal His tag (ampicillin resistance)	Novagen, USA
pETDuet-1	*E. coli* coexpression plasmid carrying an N-terminal His tag (multiple cloning site 1) or S-tag (multiple cloning site 2) (ampicillin resistance)	Novagen, USA
pBS-Ara-albaUTR-FX	Expression plasmid for *S. acidocaldarius* containing an arabinose inducible promotor and C-terminal strep tag (ampicillin resistance)	[48]
pSVAmalFX-SH10	Expression plasmid for *S. acidocaldarius* containing a maltose inducible promotor and C-terminal 10xHis tag (ampicillin resistance)	[49]
pSVAmalFX-*saci_1117*-SH10	Expression of *saci_1117* in *S. acidocaldarius*	This study
pET15b-*saci_1118*	Expression of *saci_1118* in *E. coli*	This study
pETduet1-saci_*1118*	Expression of *saci_1118* in *E. coli*	This study
pETduet1-*saci_1118-saci_1119*	Coexpression of *saci_1118, saci_1119* in *E. coli*	This study
pET15b-saci_*2032*	Expression of *saci_2032* in *E. coli*	This study
pETduet1-saci_*2032*	Expression of *saci_2032* in *E. coli*	This study
pETduet1-*saci_2032-saci_2031*	Coexpression of *saci_2031, saci_2032* in *E. coli*	This study
pBS-Ara-albaUTR-FX-*saci_2033*	Expression of *saci_2033* in *S. acidocaldarius*	This study
pSVAaraFX-HA-*saci_1119*	Expression of *saci_1119* in *S. acidocaldarius,* based on pSVAaraFX-HA	This study, [50]
pSVAaraFX-HA-*saci_2031*	Expression of *saci_2031* in *S. acidocaldarius,* based on pSVAaraFX-HA	This study
pSVA407	Gene targeting plasmid for knockout generation	[47]
pSVA12818	In-frame deletion of *saci_1117* cloned into pSVA407	This study
pSVA12822	In-frame deletion of *saci_2033* cloned into pSVA407	This study
pSVA407-Δ*saci_1494:P*$_{saci\_1117}$ *saci_1117 CtSS*	In trans complementation of MW1257, insertion of *saci_1117* with the native promoter into *saci_1494*	This study
pSVA407-Δ*saci_1494:P*$_{saci\_2033}$ *saci_2033-2034*	In trans complementation of MW1258, insertion of the *saci_2033-2034* operon with the native promoter into *saci_1494*	This study

Supplementary Table 7 Sequences of oligonucleotides used in this study. Restriction sites are marked in red and underlined. GA, Gibson Assembley.

ORFs	Name	Sequences (5´→3´)
**Oligonucleotides for cloning**		
Saci_1117	*Fw (pSVAmalFX-SH10)-NcoI*	CAAGATCCATGGTGTCCAAATACATATTGGC
	Rev (pSVAmalFX-SH10)-XhoI	CTGCTTCTCGAGTTCCATAGTCTCCACTTC
	Fw (pSVA407) upstream	CCCGGATCCGCTTACCCTCTAGGCTTTCC
	Rev (pSVA407) upstream	CAAATACATAATGGAATAGAGAAATGCCAAAAG
	Fw (pSVA407) downstream	TCTATTCCATTATGTATTTGGACACATTAACTATTTT
	Rev (pSVA407) downstream	AGCCGGGCCCCTCAAGACCCTCTTCACTTC
	Fw (pSVA407comp) GA	GCAGAGTATTTTGTAGGTCCAGTGACCTCTTTCTACTAAA GTAACG
	Rev (pSVA407comp) GA	CTGTCTCGAATTCCGTTCTCCTTACTTTTCGAACTGTGGG TGACTC
Saci_1118	*Fw (pET15b)-NdeI*	GTCCGACATATGATGAAGCAAAACAGTTCAG
	Rev (pET15b)-BamHI	AATTACGGATCCTTATTCCCCCCTCGCCAG
	Fw (pETDuet-1)-BamHI	GAGGAGGGATCCATGAAGCAAAACAGTTCAG
	Rev (pETDuet-1)-HindIII	GAGGAGAAGCTTTTATTCCCCCCTCGCCAG
Saci_1119	*Fw (pETDuet-1)-NdeI*	GAGGAGCATATGATGGAGATGAGTGGGAAG
	Rev (pETDuet1)-XhoI	GAGGAGCTCGAGTTAAACTCTTGAAAGACAGGAGA
	*Fw (pSVaraFX-HA)-Nco*I	CATCCATGGAGATGAGTGGGAAGTT
	*Rev (pSVaraFX-HA)-Xho*I	CATCTCGAGAACTCTTGAAAGACAGGAGA
Saci_2031	*Fw (pETDuet-1)-NdeI*	GAGGAGCATATGATGGAGATCAGCGGAGAG
	Rev (pETDuet1)-XhoI	GAGGAGCTCGAGCTAGGTAACTCTAGAAC
	*Fw (pSVaraFX-HA)-Nco*I	CATCCATGGAGATCAGCGGAGAGTTTG
	*Rev (pSVaraFX-HA)-Xho*I	CATCTCGAGGGTAACTCTAGAACGTATACAG
Saci_2032	*Fw (pET15b)-NdeI*	GTCCGACATATGATGGAAATAAAAACAAGCG
	Rev (pET15b)-BamHI	AATTACGGATCCTCATCTCCCTCTTGCAATTAATG
	Fw (pETDuet-1)-BamHI	GAGAGGGATCCATGGAAATAAAAACAAGCG
	Rev (pETDuet-1)-HindIII	GAGAGAAGCTTTCATCTCCCTCTTGCAATTAATG
Saci_2033	*Fw (pBS-Ara-albaUTR-FX)-NcoI*	GAACCATGGTGGCTGAAAAATACGTGATAG
	Rev (pBS-Ara-albaUTR-FX)-XhoI	GGTCTCGAGACCCCCAATAGTCTTAGC
	Fw (pSVA407) upstream	GCATCTCGAGCCTGTATCTCTTATAGCGTG
	Rev (pSVA407) upstream	TACAACTTCCGATGCCATAGCATTTTATCCATTTCTATTT
	Fw (pSVA407) downstream	ATAGAAATGGATAAAATGCTATGGCATCGGAAGTTGTAAG
	Rev (pSVA407) downstream	GCATGGGCCCTGATTAACTTCAGAAAACAGTG
	Fw (pSVA407comp) GA	GCAGAGTATTTTGTAGGTCCAGTGAGCTATGTTTATTGTT TCCACTTTTC
	Rev (pSVA407comp) GA	TTCACTGTCTCGAATTCCGTTCTCCCTTTAGTGGAGACAC CTGTTGGAG
Saci_1494 up	*Fw (pSVA407comp) GA*	ACGGCCAGTGAATTGTAATACGACTCACTATAGGGCGAAT TGGGCCGTGTATAATGATGACCTATTTAGCTG
	Rev (pSVA407comp1117) GA	GTTACTTTAGTAGAAAGAGGTCACTGGACCTACAAAATAC TCTG
	Rev (pSVA407comp2033) GA	GAAAAGTGGAAACAATAAACATAGCTCACTGGACCTACAA AATACTCTG
Saci_1494 down	*Fw (pSVA407comp1117) GA*	GTCACCCACAGTTCGAAAAGTAAGGAGAACGGAATTCGA GACAGTG

Fw (pSVA407comp2033) GA	ACTCCAACAGGTGTCTCCACTAAAGGGAGAACGGAATTC GAGACAGTGA
Rev (pSVA407comp) GA	AGGCGGCCGCGAATTCACTAGTGATATCGAATTCCCGCC CTCTGATAATTCAGTGGCTTTATTC

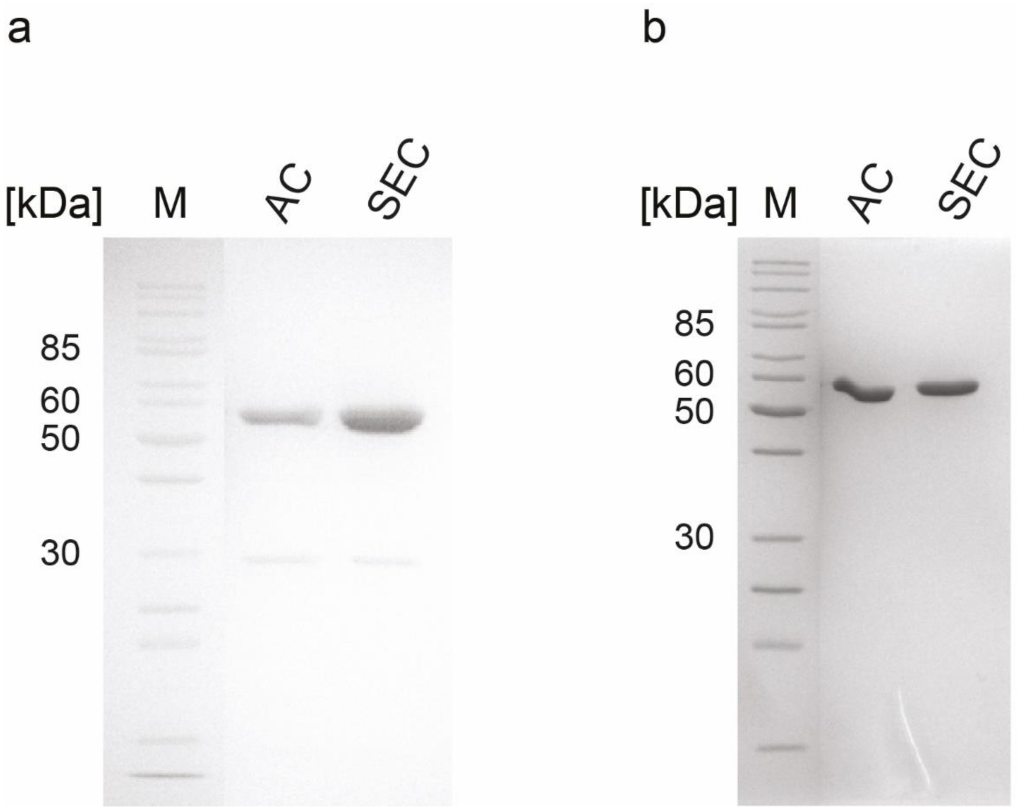

Supplementary Fig. 1 Purification of recombinant glycerol kinase isoenzymes Saci_1117 and Saci_2033. The glycerol kinases (GK) Saci_1117 (**a**) and Saci_2033 (**b**) were homologously produced in *S. acidocaldarius* MW001 using the pSVAmalFX-SH10 and pBS-Ara-albaUTR-FX expression vector, respectively. Proteins were purified via His-tag (Saci_1117) and Strep-tag (Saci_2033) affinity chromatography (AC), respectively, and size exclusion chromatography (SEC). Proteins (2 µg) were separated via SDS-PAGE and stained via Coomassie Blue. Marker (M): prestained PageRuler™ (Thermo Fischer Scientific, USA).

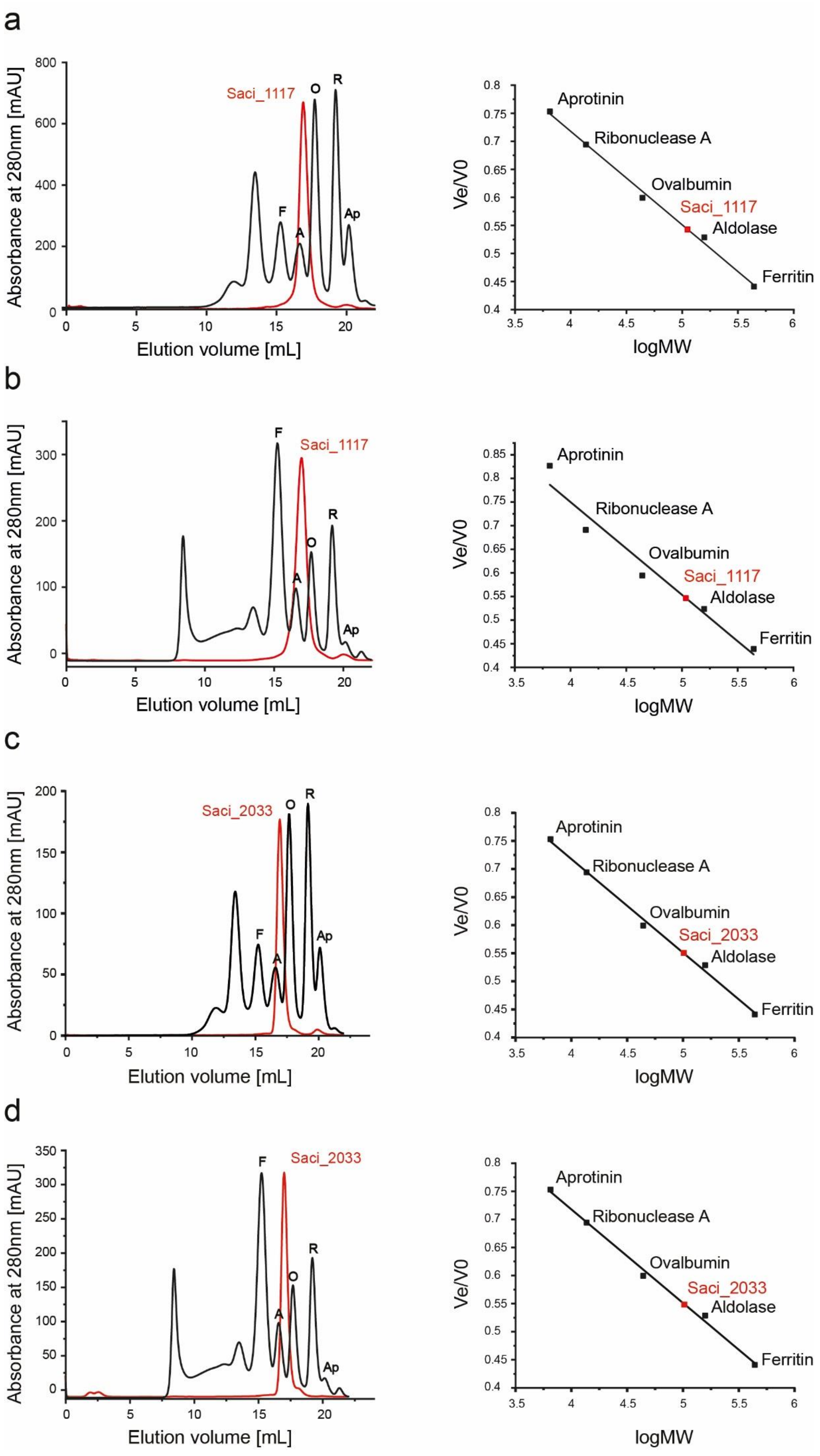
a
Absorbance at 280nm [mAU]
Elution volume [mL]
Saci_1117
O
R
F
A
Ap
Ve/V0
logMW
Aprotinin
Ribonuclease A
Ovalbumin
Saci_1117
Aldolase
Ferritin
b
Absorbance at 280nm [mAU]
Elution volume [mL]
F
Saci_1117
R
O
A
Ap
Ve/V0
logMW
Aprotinin
Ribonuclease A
Ovalbumin
Saci_1117
Aldolase
Ferritin
c
Absorbance at 280nm [mAU]
Elution volume [mL]
Saci_2033
O
R
F
A
Ap
Ve/V0
logMW
Aprotinin
Ribonuclease A
Ovalbumin
Saci_2033
Aldolase
Ferritin
d
Absorbance at 280nm [mAU]
Elution volume [mL]
F
Saci_2033
R
O
A
Ap
Ve/V0
logMW
Aprotinin
Ribonuclease A
Ovalbumin
Saci_2033
Aldolase
Ferritin

Supplementary Fig. 2 Size exclusion chromatography of the recombinant GK isoenzymes Saci_1117 and Saci_2033 from *S. acidocaldarius.* Recombinant proteins were separated on a Superose 6 10/300 column in both, the absence ((**a**), Saci_1117 and (**c**), Saci_2033) and presence of 10 mM glycerol ((**b**), Saci_1117 and (**d**), Saci_2033). In the elution profiles (left panels) the GKs Saci_1117 and Saci_2033 from *S. acidocaldarius* are shown in red and the standard proteins for calibration in black. Abbreviations represent the following marker proteins of the Gel Filtration Calibration Kits (LMW, HMW; Cytiva) (F) – Ferritin, (A) – Aldolase, (O) – Ovalbumin, (R) – Ribonuclease A, (Ap) – Aprotinin. The respective calibration curves are shown in the right panels.

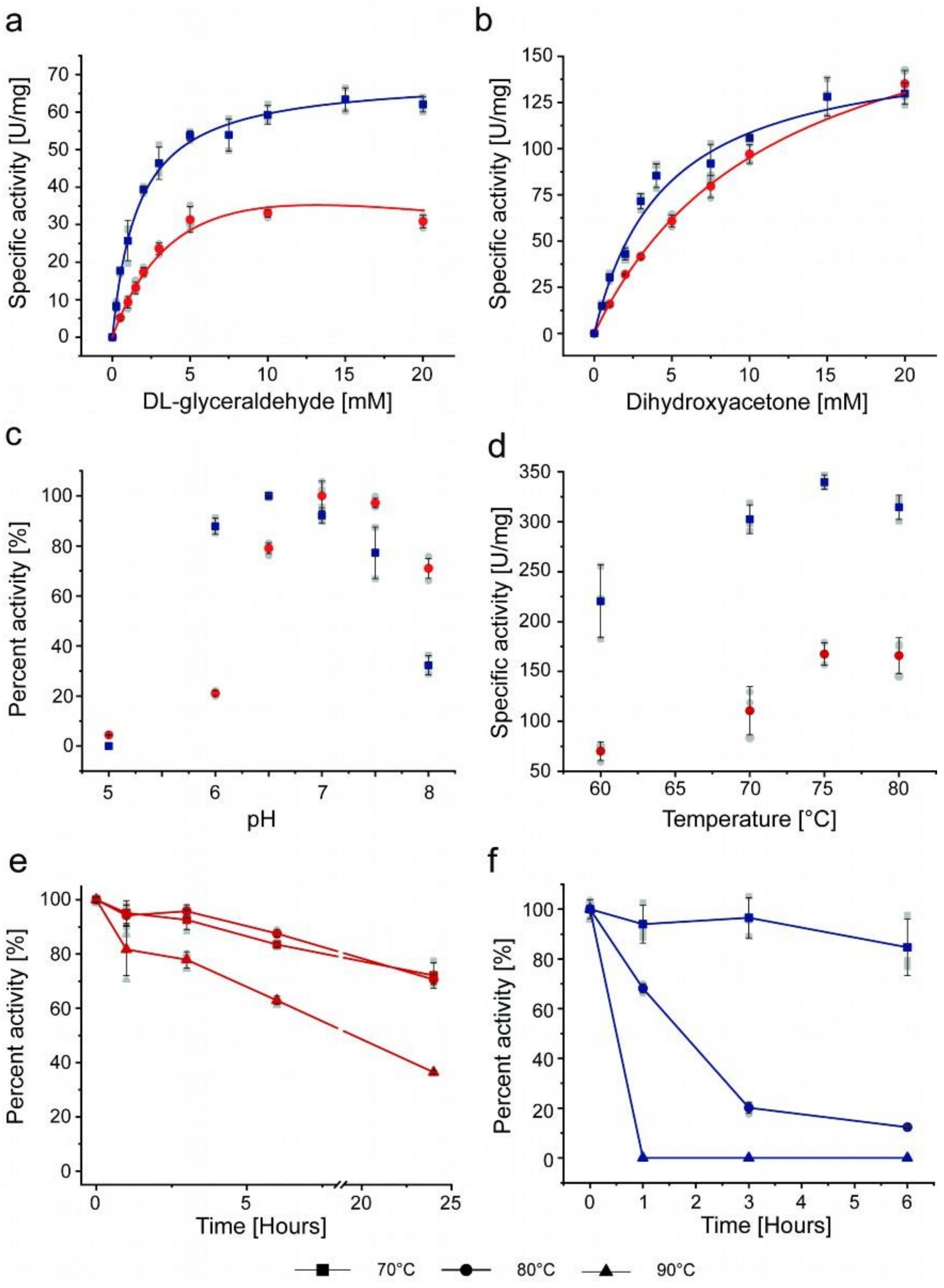

a
b
c
d
e
f
Specific activity [U/mg]
DL-glyceraldehyde [mM]
Dihydroxyacetone [mM]
Percent activity [%]
pH
Temperature [°C]
Time [Hours]
70°C
80°C
90°C

Supplementary Fig. 3 Biochemical characterization of recombinant GK isoenzymes Saci_1117 and Saci_2033. The kinetic properties of Saci_1117 (red circle) and Saci_2033 (blue square) with (**a**) DL-glyceraldehyde and dihydroxyacetone (**b**) as substrate were determined using the continuous PK-LDH assay at 50 °C in 0.1 M TRIS-HCl pH 7 for Saci_1117 and 0.1 M MOPS pH 6.5 for Saci_2033, respectively. (**c**) The pH optimum of the Saci_1117 (red circle) and Saci_2033 (blue square) was determined in the range of pH 5.0-8.0 using the continuous PK-LDH assay in a mixed buffer system (0.05 M MES, 0.05 M HEPES, 0.05 M TRIS) at 50°C. (**d**) The temperature optimum of Saci_1117 (red circle) and Saci_2033 (blue square) was determined between 60 °C to 80 °C using a continuous assay with G3PDH (Saci_2032) as auxiliary enzyme and 0.1 mM DCPIP as electron acceptor. The thermal stability of the GK Saci_1117 (**e**) and Saci_2033 (**f**) was determined by monitoring the residual activity over time (1, 3, 6, 24 h) at 70, 80 and 90°C. The corresponding symbols for each temperature condition are provided below. Saci_1117 was incubated in 100 mM TRIS-HCl, pH 7 (temperature adjusted) and Saci_2033 in 100 mM MOPS-KOH, pH 6.5 (temperature adjusted) at a protein concentration of 0.05 mg ml^{-1}. Residual activity was determined using the continuous PK-LDH assay at 50°C. All experiments were performed in triplicate (n=3, technical replicates) and error bars indicate the standard deviation (SD) of the mean and individual data points are shown as grey dots.

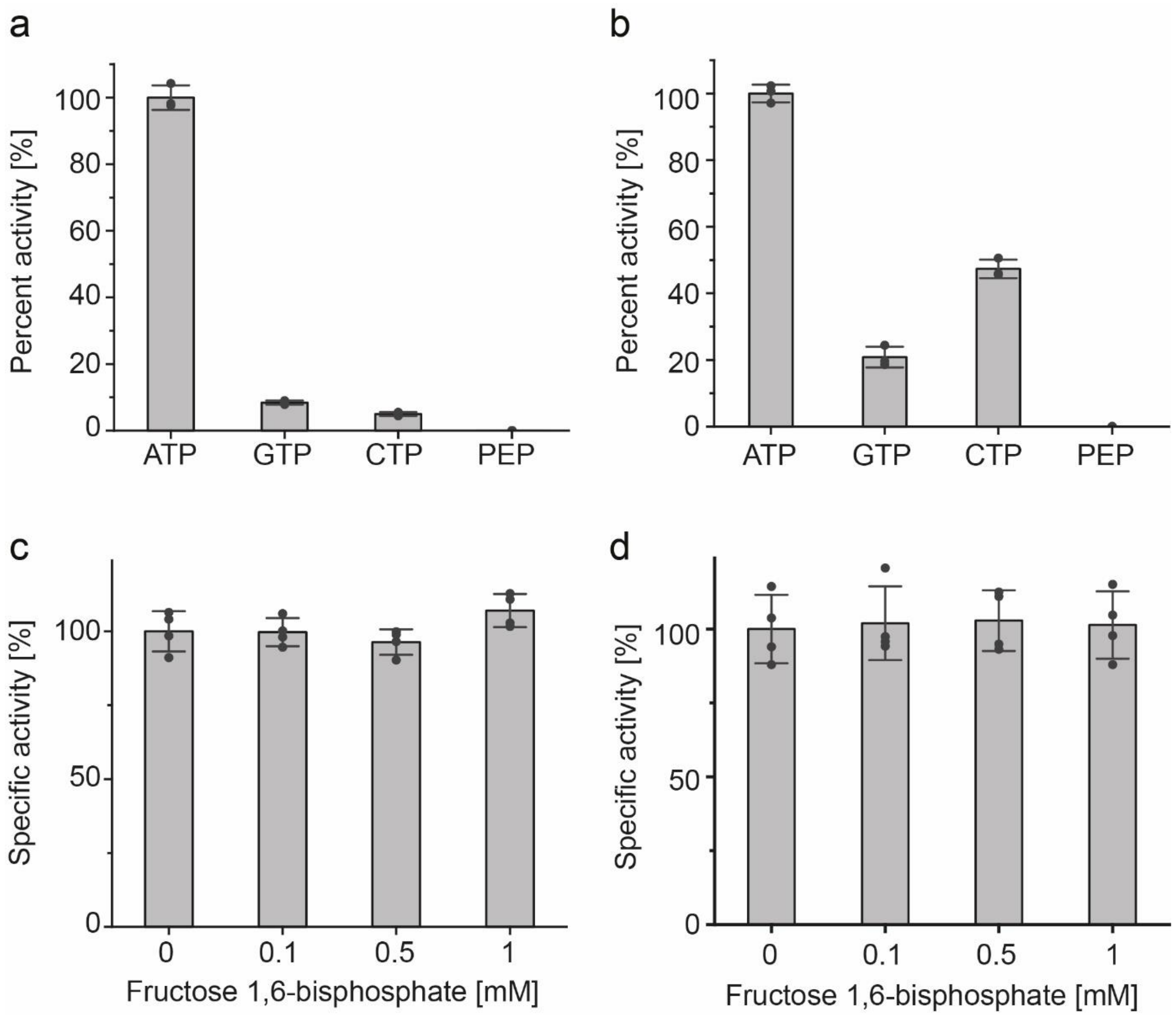

Supplementary Fig. 4 Phosphate donor specificity and effect of fructose 1,6-bisphosphate on enzyme activity of the recombinant GK isoenzymes Saci_1117 and Saci_2033. The enzyme activity of Saci_1117 (**a**) and Saci_2033 (**b**) with 2 mM glycerol as substrate and 5 mM of different phosphate donors was determined in a continuous assay at 75 °C using G3PDH Saci_2032 as auxiliary enzyme. G3PDH couples the phosphorylation of glycerol by GK to the oxidation of G3P to DHAP by following the decrease in absorbance at 600 nm due to the reduction of DCPIP. The effect of fructose 1,6-bisphosphate on enzyme activity of Saci_1117 (**c**) and Saci_2033 (**d**) was determined in presence of increasing fructose 1,6-bisphosphate concentrations using the continuous coupled LDH-PK assay at 50°C. All values represent the average of three (**a,b**) or four (**c**,**d**) independent measurements (technical replicates). Error bars represent the SD of the mean and individual data points are shown as dots.

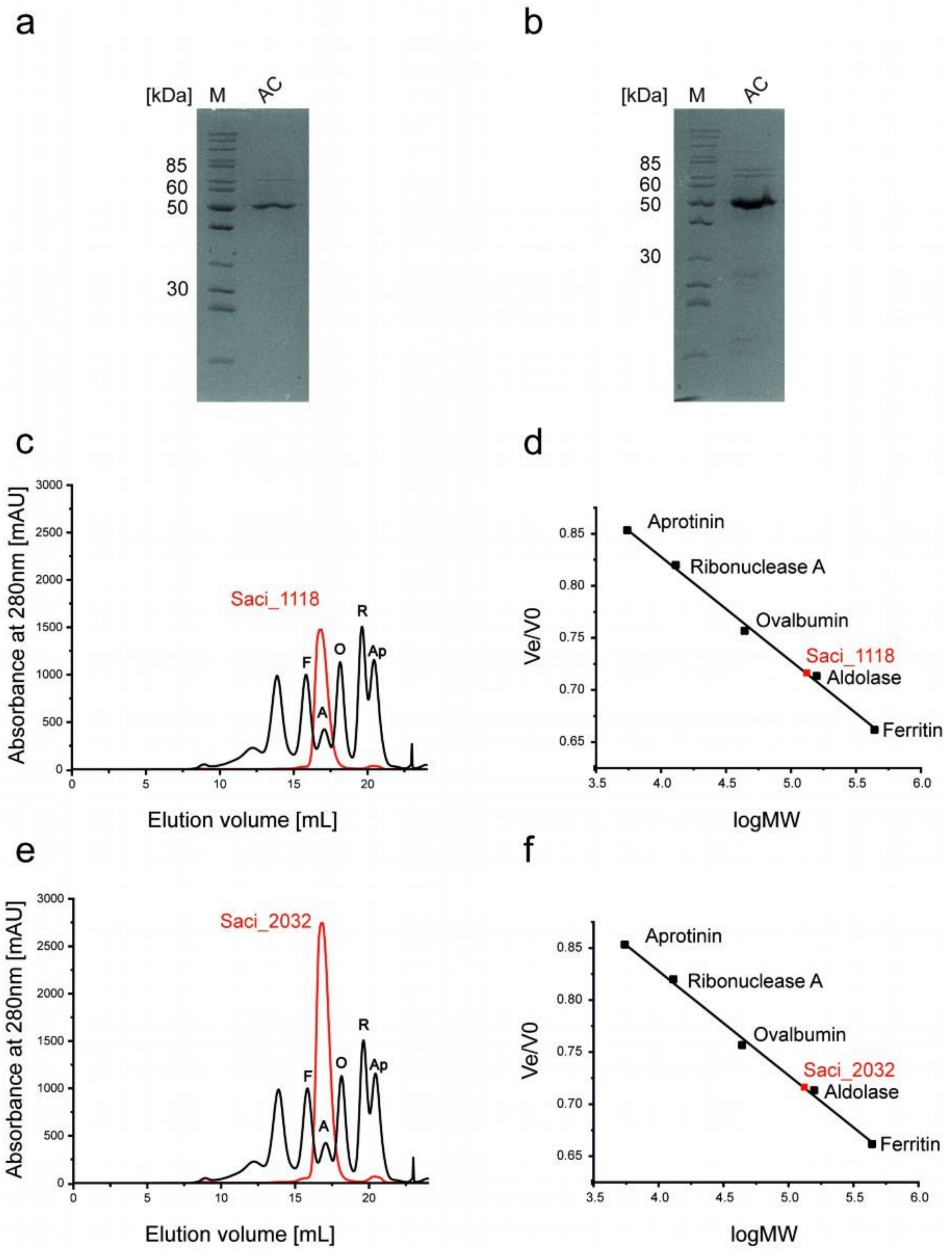
a
[kDa] M AC
85
60
50
30
b
[kDa] M AC
85
60
50
30
c
Absorbance at 280nm [mAU]
Elution volume [mL]
Saci_1118
F
A
O
R
Ap
d
Ve/V0
logMW
Aprotinin
Ribonuclease A
Ovalbumin
Saci_1118
Aldolase
Ferritin
e
Absorbance at 280nm [mAU]
Elution volume [mL]
Saci_2032
F
A
O
R
Ap
f
Ve/V0
logMW
Aprotinin
Ribonuclease A
Ovalbumin
Saci_2032
Aldolase
Ferritin

Supplementary Fig. 5 Purification and size exclusion chromatography of the recombinant G3PDH isoenzymes Saci_1118 and Saci_2032 from *S. acidocaldarius.* The glycerol-3-phosphate dehydrogenases (G3PDH) Saci_2032 (**a**) and Saci_1118 (**b**) were heterologously produced in *E. coli* Rosetta using pET15b and purified via His-tag affinity chromatography (AC). Proteins (2 µg) were separated via SDS-PAGE and stained via Coomassie Blue. Marker (M): prestained PageRuler™ (Thermo Fischer scientific, USA). Recombinant proteins, (**c, d**), Saci_1118 and (**e, f**), Saci_2032, were separated on a Superose 6 10/300 column. In the elution profiles (left panels) the G3PDHs Saci_1118 and Saci_2032 from *S. acidocaldarius* are shown in red and the standard proteins for calibration in black. Abbreviations represent the following marker proteins of the Gel Filtration Calibration Kits (LMW, HMW; Cytiva) (F) – Ferritin, (A) – Aldolase, (O) – Ovalbumin, (R) – Ribonuclease A, (Ap) – Aprotinin. The respective calibration curves are shown in the right panels.

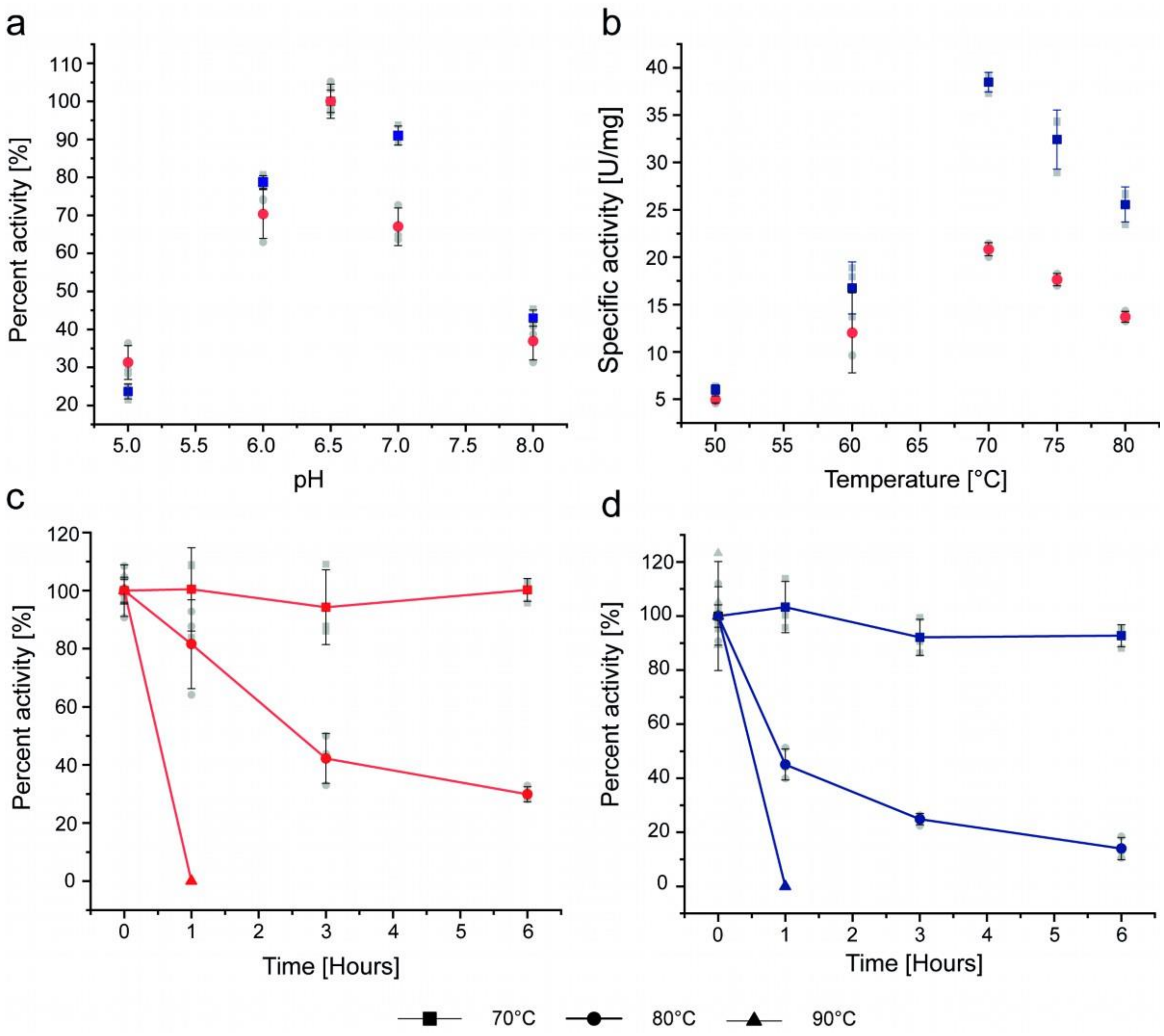

Supplementary Fig. 6 Biochemical characterization of recombinant G3PDH isoenzymes Saci_1118 and Saci_2032. (**a**) The pH dependence of Saci_1118 (red circle) and Saci_2032 (blue square) was determined in a continuous assay as G3P-dependent reduction of DCPIP in a mixed buffer system (0.05 M MES, 0.05 M HEPES, 0.0 5M TRIS) at 70°C. (**b**) The optimum temperature of Saci _1118 and Saci_2032 was determined in the same assay system in 0.05 M MES-KOH pH 6.5 adjusted between 60 °C to 80 °C. (**c**) Heat stability of Saci_1118 (**c**) and Saci_2032 (**d**) were determined by monitoring the residual activity upon incubation at 70, 80 and 90 °C for up to 6 hours. The corresponding symbols for each temperature condition are provided below. The enzymes (protein concentrations of 0.32 mg ml^{-1} for Saci_1118 and 0.18 mg ml^{-1} for Saci_2032) were incubated in 50 mM MES-KOH, pH 6.5 (temperature adjusted) and after the respective incubation times the residual G3PDH activity was determined with DCPIP as electron acceptor at 70°C. Experiments were performed in triplicate (n=3, technical replicates) and error bars indicate the SD of the mean and individual data points are shown as grey dots.

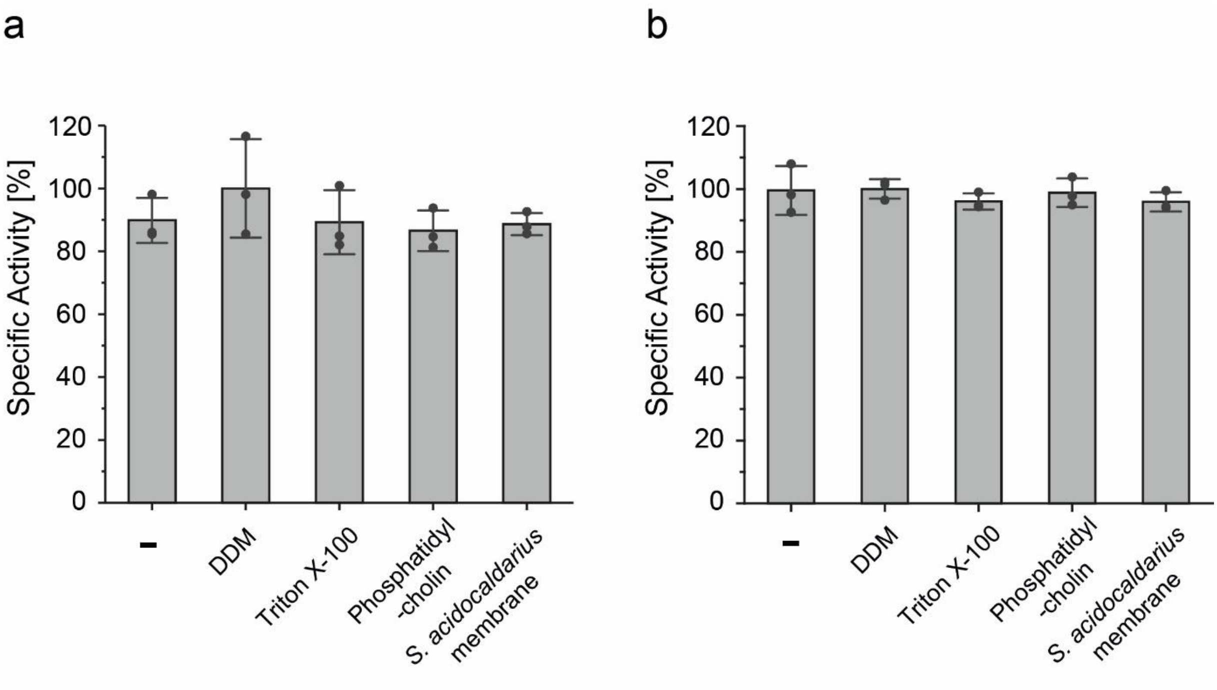

Supplementary Fig. 7 Effect of non-ionic detergents and phospholipids on the activity of recombinant G3PDH isoenzymes Saci_1118 and Saci_2032. The activity of Saci_1118 (**a**) and Saci_2032 (**b**) in the presence of potential activators was determined in a continuous assay by coupling the oxidation of G3P to the reduction of DCPIP in a MES-KOH buffer pH 6.5 at 50°C. The effect of 0.5% (w/v) DDM, 0.5% (v/v) of triton X-100, 50 µg of phosphatidylcholine or 50 µg of isolated *S. acidocaldarius* membrane fractions was tested. All measurements were performed in triplicate (n=3, technical replicates). Error bars represent the SD of the mean and individual data points are shown as dots.

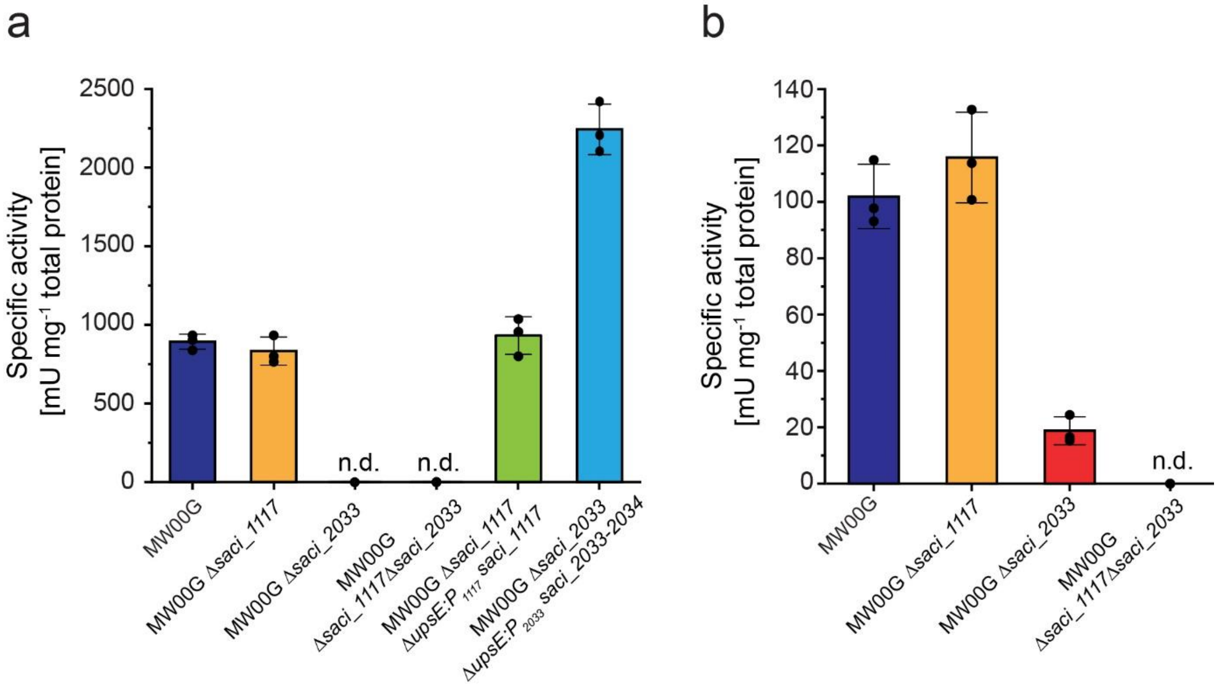

Supplementary Fig. 8 Comparison of glycerol kinase (GK) activities in *S. acidocaldarius* parental (MW00G) and GK deletion strains. (**a**) GK activity measured in crude extracts from glycerol grown cells of the parental MW00G, single deletion strains Δ*saci_1117* and Δ*saci_2033*, the double deletion strain Δ*saci_1117*Δ*saci_2033*, and the single deletion strains Δ*saci_1117* and Δ*saci_2033*, complemented *in trans* by ectopic integration of the wildtype genes *saci_1117* and the *saci_2033-34* operon, respectively. n.d. indicates activity was not detected due to growth deficiencies on glycerol. (**b**) GK activity determined in crude extracts from N-Z-amine grown cells of the parental MW00G, single Δ*saci_1117* and Δ*saci_2033* and double Δ*saci_1117*Δ*saci_2033* GK deletion mutants. n.d. indicates that activity could not be detected. All measurements were performed in triplicate (n=3, technical replicates). Error bars represent the SD of the mean and individual data points are shown as dots.

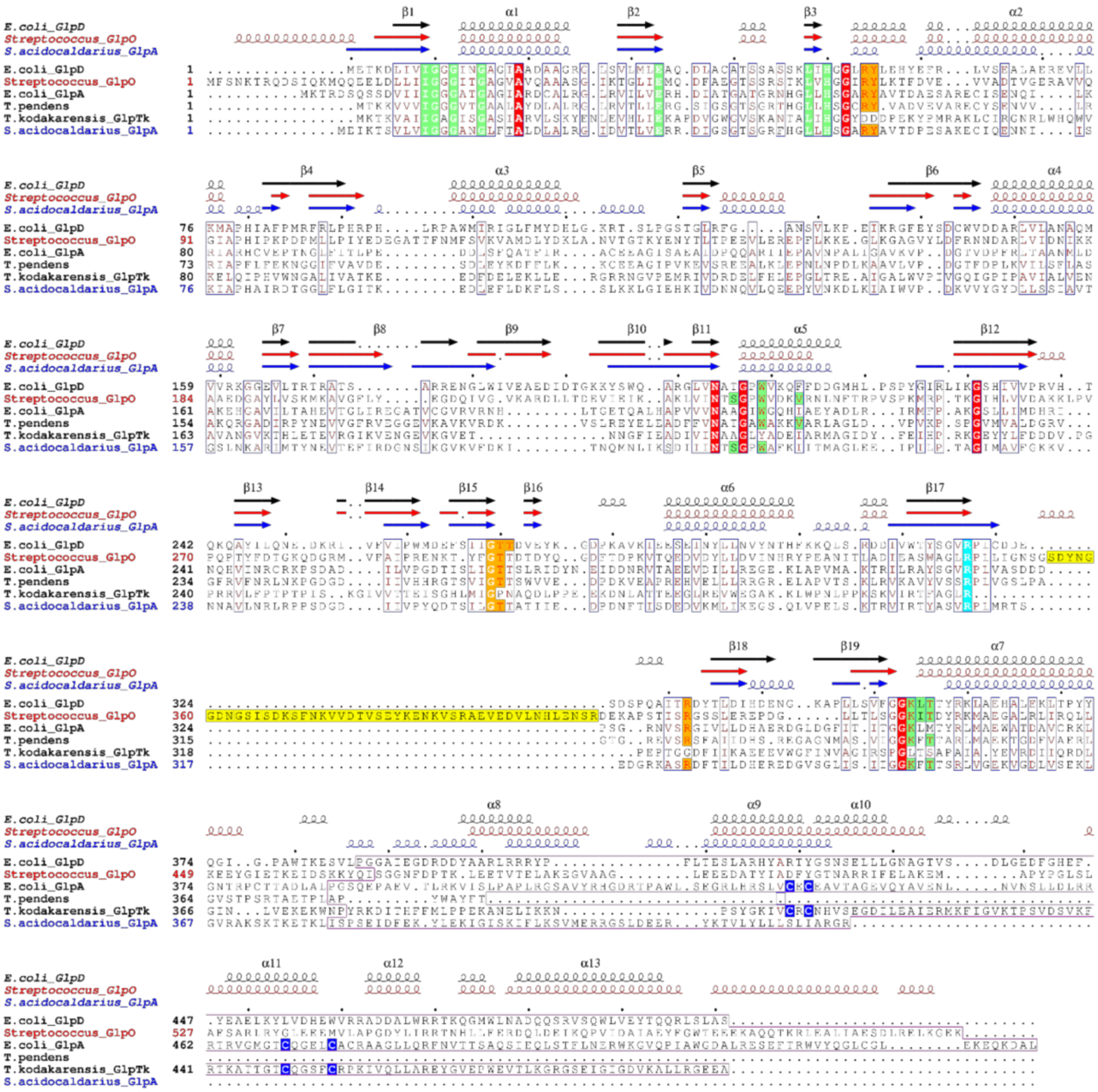

Supplementary Fig. 9 Sequence alignment of *S. acidocaldarius* G3PDH (Saci_2032) with *E. coli* GlpD (2qcu, P13035) and GlpA (P0A9C0), GlpO from *Streptococcus* sp. (2rgo, D0VWP7) as well as archaeal homologues from *T. pendens* (A1RZ95, Tpen_1127) and *T. kodakarensis* (Q5JGZ7, TK1393). The secondary structural elements from the available crystal structures of GlpD and GlpO as well as the predicted structure for *S. acidocaldarius* G3PDH by alphaFold based modelling are depicted above of the sequences. The alignment illustrates the sequence and structural conservation in the N-terminal 370-390 aa residues. The residues shown to be involved in FAD cofactor binding and substrate (G3P/DHAP) binding in the GlpD and GlpO sturctures as well as their conservation in the other sequences are shaded green and orange, respectively. The arginine residue supposed to act as general base in catalysis is highlighted in cyan. The sequence insertion distinctive for GlpOs is shaded yellow. The amino acid residues comprising the C-terminal domains are boxed in pink with the cysteine residues forming the FeS clusters in GlpA and the *Thermococcus* G3PDH marked by blue shading. Sequences were aligned with clustal W implemented in the MEGA 11 software package. The figure was generated using ESPript 3.0 (Robert and Gouet, 2014).

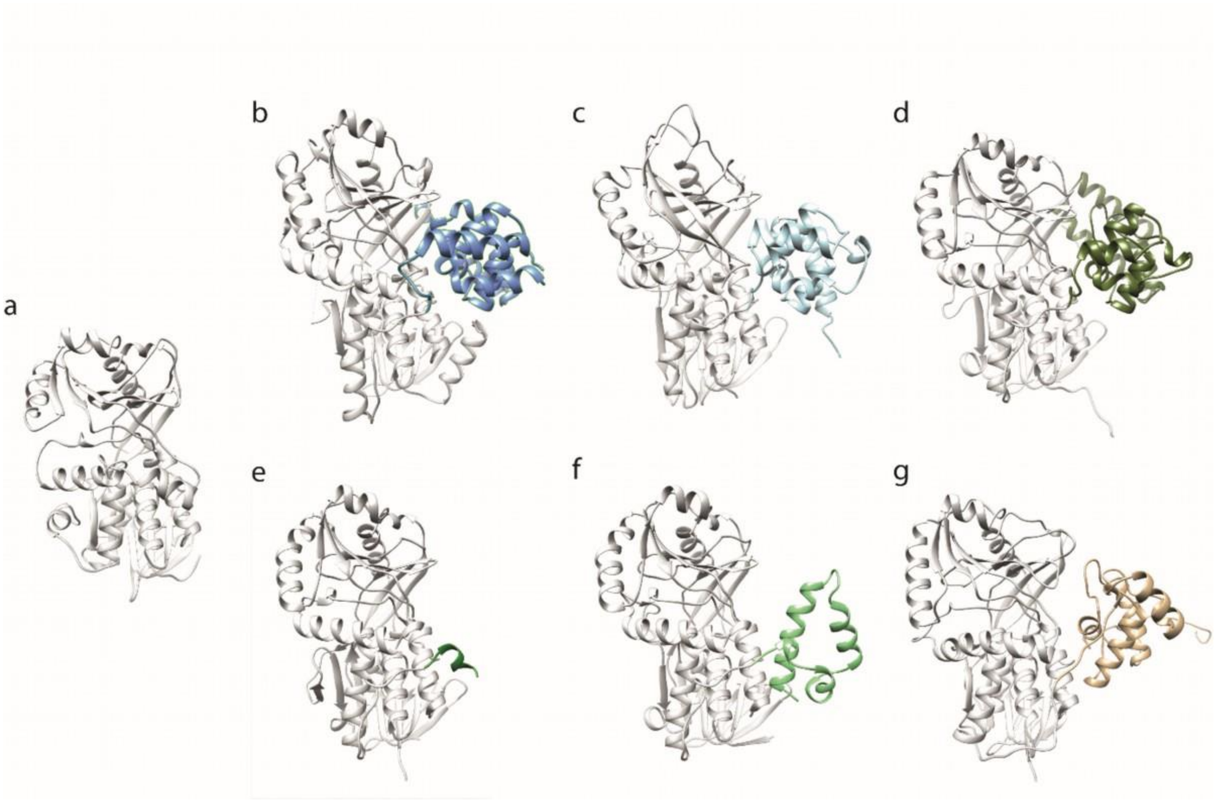

Supplementary Fig. 10 Comparison of the overall fold of the catalytic subunits of the different types of FAD dependent G3P oxidizing enzymes. The ribbon representations of crystal structures of (**a**) the *B. subtilis* glycine oxidase (1ng3) (Settembre et al., 2003) comprising the DAAO fold lacking any C-terminal extensions (shown in gray in all structures) is comparatively illustrated with those of (**b**) GlpO (2rgo) (Colussi et al., 2008), (**c**) GlpD (2qcu) (Yeh et al., 2008b), as well as the structural models (generated using CoLabFold (Mirdita et al., 2022)) of (**d**) *E. coli* GlpA, (**e**) the GlpA homologues of *T. pendens* and (**f**) *S. acidocaldarius*, and (g) the GlpTk from *T. kodakarensis* (TK1393). The C-terminal domains are highlighted using the same colour code as in Fig. 9-10 in the main text to emphasize the differences in size and fold.

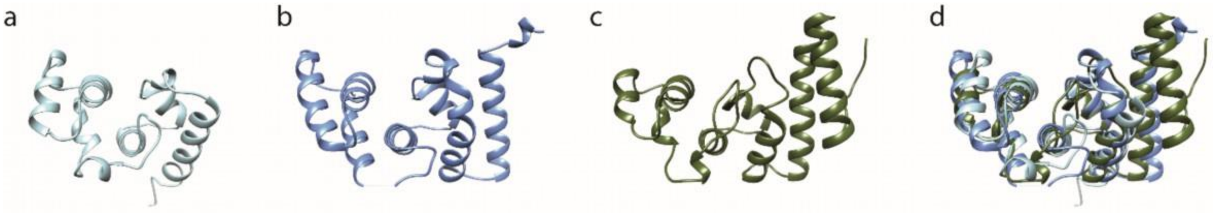

Supplementary Fig. 11 Comparison of C-terminal domains of GlpD (a), GlpO (b), and GlpA (c). The crystal structures of the C-termini of GlpD from *E. coli* (2qcu) (**a**) and GlpO from *Streptococcus* sp. (2rgo) (**b**) are shown, as well as the structural model of the C-terminus of GlpA from *E. coli* (**c**). The comparison reveals that the GlpD C-terminal fold is also present in GlpO and GlpA but extended by one and two helices, respectively. In (**d**) the superimposition of the three structures is shown in the same orientation as in (**a**)-(**c**). The dashed line in (**b**) indicates missing structural information for M518 in the C-terminus of *Streptococcus* sp. GlpO.

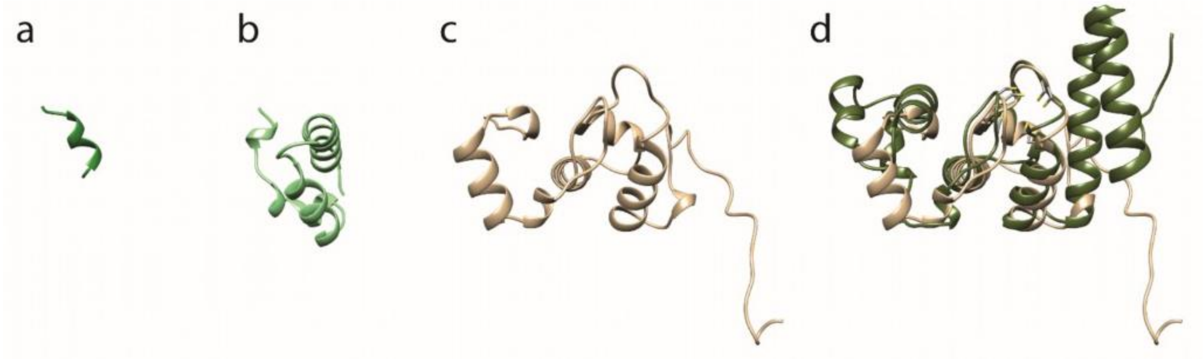

Supplementary Fig. 12 Comparison of C-terminal extensions from GlpA-like G3PDHs from *T. pendens* (a) and *S. acidocaldarius* (Saci_2032, b), and of the GlpTk from *T. kodakarensis* (TK1393, c). The ribbon representation of the structural models of the C-terminal are shown. To illustrate the presence of the bfd-fold in GlpTk and GlpA a superimposition of both C-termini is presented (**d**). Both structures match in their central parts as well as in the four conserved cysteine residues shown as stick models whereas the other parts of the structure differ remarkably.

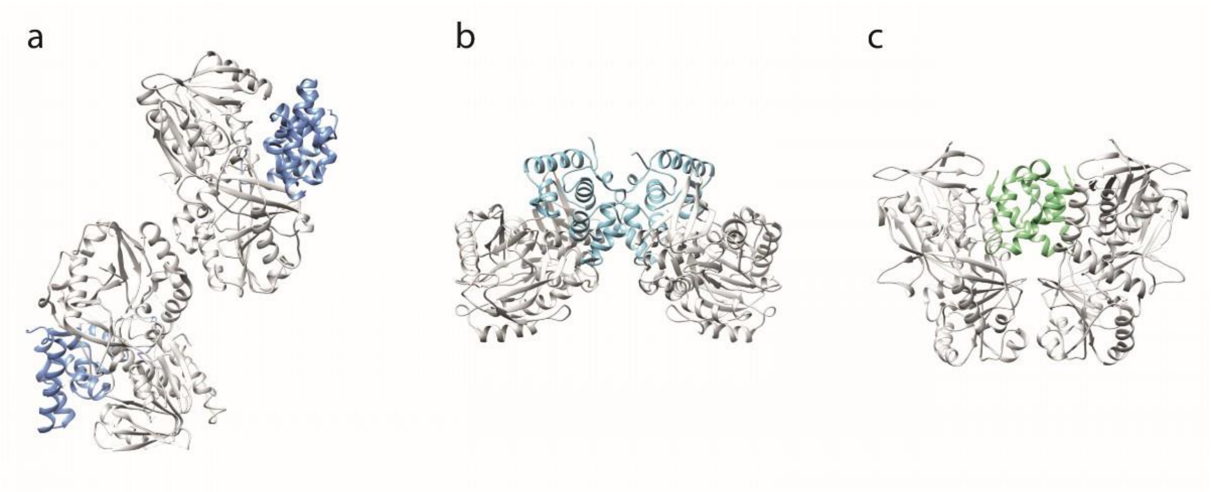

Supplementary Fig. 13 Comparison of the dimer formation in the crystal structures of the (a) soluble, cytoplasmic GlpO (*Streptococcus*. spp., 2rgo) and (b) the monotopic, membrane bound GlpD (*E. coli*, 2qcu) with (c) the structural model of the GlpA-like Saci_2032 dimer. The three structures illustrate the different role of the C-terminus (coloured according to the preceeding figures) in dimer formation as well as the resulting different spatial orientation of the DAAO-folds of the subunits to each other.

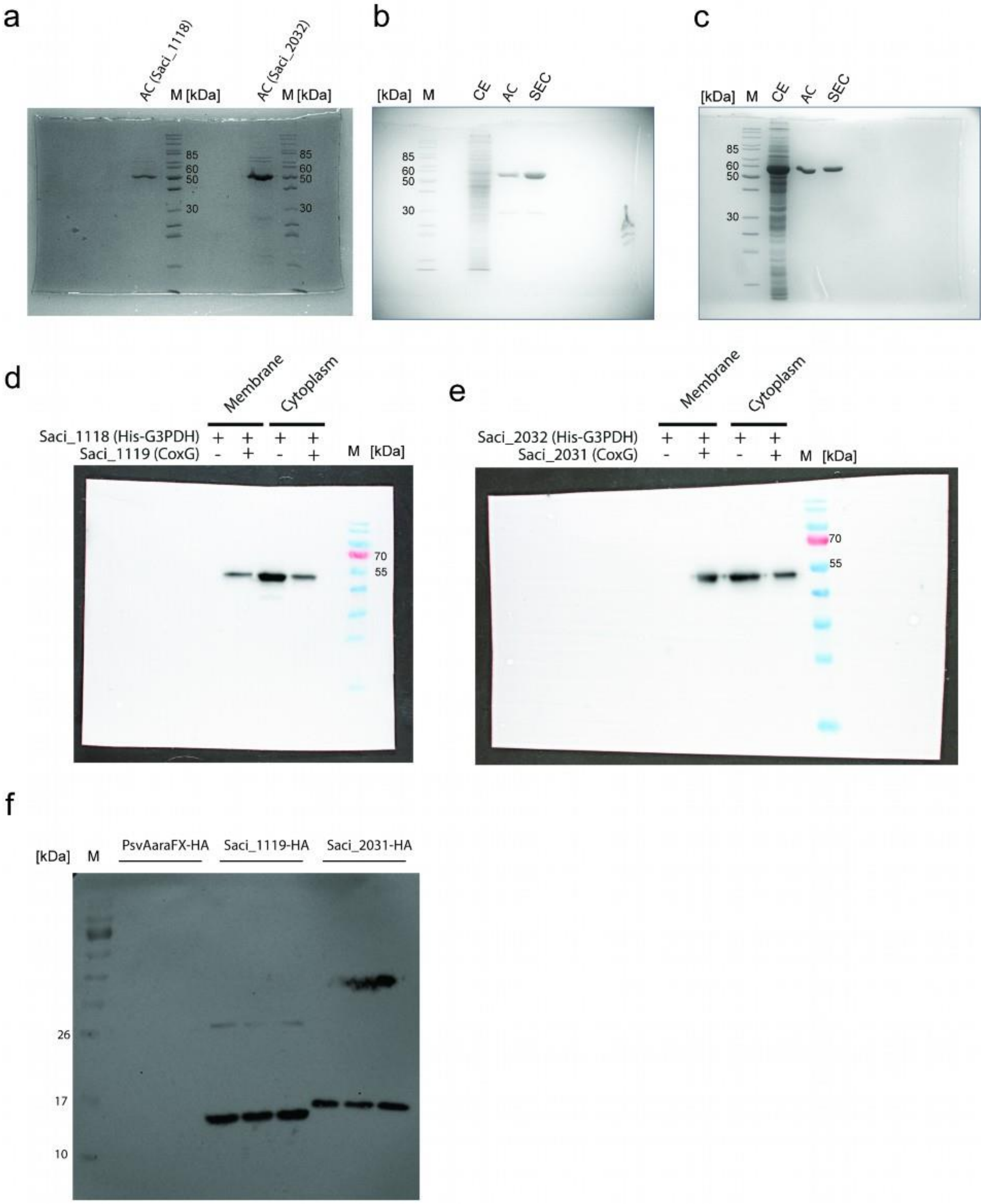
a
AC (Saci_1118)
M [kDa]
AC (Saci_2032)
M [kDa]
85
60
50
30
85
60
50
30
b
[kDa] M
CE
AC
SEC
85
60
50
30
c
[kDa] M
CE
AC
SEC
85
60
50
30
d
Membrane
Cytoplasm
Saci_1118 (His-G3PDH) + + + +
Saci_1119 (CoxG) - + - +
M [kDa]
70
55
e
Membrane
Cytoplasm
Saci_2032 (His-G3PDH) + + + +
Saci_2031 (CoxG) - + - +
M [kDa]
70
55
f
[kDa] M
PsvAaraFX-HA
Saci_1119-HA
Saci_2031-HA
26
17
10

Supplementary Fig. 14: Uncropped version of gels and Western blots. (**a)**The glycerol-3-phosphate dehydrogenases (G3PDH) Saci_2032 and Saci_1118 were heterologously produced in *E. coli* Rosetta using pET15b and purified via His-tag affinity chromatography (AC). The glycerol kinases (GK) Saci_1117 (**b**) and Saci_2033 (**c**) were homologously produced in *S. acidocaldarius* MW001 using the pSVAmalFX-SH10 and pBS-Ara-albaUTR-FX expression vector, respectively. Proteins were purified via His-tag (Saci_1117) and Strep-tag (Saci_2033) affinity chromatography (AC), respectively, and size exclusion chromatography (SEC). The effect of heterologous co-expression of CoxGs on the localization of His-tagged G3PDHs in the cytoplasmic and membrane fractions of *E. coli* was analyzed via western blotting, followed by immunodetection using an anti-His antibody for Saci_1118/Saci_1119 (**d**) and Saci_2031/Saci_2032 (**e**). Isolated membrane fractions from *S. acidocaldarius* cells following homologous overexpression of HA-tagged CoxG homologs Saci_1119 and Saci_2031 utilizing the pSVAaraFX-HA vector, were analyzed (**f**).

Supplementary references

1. Lu S*, et al.* CDD/SPARCLE: the conserved domain database in 2020. *Nucleic acids research* **48**, D265-D268 (2020).

2. Schnick C*, et al.* Structure and non-essential function of glycerol kinase in *Plasmodium falciparum* blood stages. *Molecular Microbiology* **71**, 533-545 (2009).

3. Balogun EO*, et al.* Biochemical characterization of highly active *Trypanosoma brucei gambiense* glycerol kinase, a promising drug target. *The journal of biochemistry* **154**, 77-84 (2013).

4. Wilk P*, et al.* Structural characterization of glycerol kinase from the thermophilic fungus *Chaetomium thermophilum*. *International Journal of Molecular Sciences* **21**, 9570 (2020).

5. Fukuda Y*, et al.* Epistasis effects of multiple ancestral-consensus amino acid substitutions on the thermal stability of glycerol kinase from *Cellulomonas* sp. NT3060. *Journal of bioscience and bioengineering* **121**, 497-502 (2016).

6. Thorner JW, Paulus H. Glycerol and glycerate kinases. In: *The enzymes*. Elsevier (1973).

7. Huang H-S*, et al.* Purification and characterization of thermostable glycerol kinase from *Thermus flavus*. *Journal of fermentation and bioengineering* **83**, 328-332 (1997).

8. Sakasegawa S-i*, et al.* A novel glycerol kinase from *Flavobacterium meningosepticum*: characterization, gene cloning and primary structure. *Bioscience, biotechnology, and biochemistry* **62**, 2388-2395 (1998).

9. Hokao R*, et al.* Affinity shift of ATP upon glycerol binding to a glycerol kinase from the hyperthermophilic archaeon *Thermococcus kodakarensis* KOD1. *Journal of Bioscience and Bioengineering* **129**, 657-663 (2020).

10. Applebee MK, Joyce AR, Conrad TM, Pettigrew DW, Palsson BØ. Functional and metabolic effects of adaptive glycerol kinase (GLPK) mutants in *Escherichia coli*. *Journal of Biological Chemistry* **286**, 23150-23159 (2011).

11. Pawlyk AC, Pettigrew DW. Subcloning, expression, purification, and characterization of *Haemophilus influenzae* glycerol kinase. *Protein expression and purification* **22**, 52-59 (2001).

12. Balogun EO*, et al.* Molecular basis for the reverse reaction of African human trypanosomes glycerol kinase. *Molecular microbiology* **94**, 1315-1329 (2014).

13. Koga Y, Katsumi R, You D-J, Matsumura H, Takano K, Kanaya S. Crystal structure of highly thermostable glycerol kinase from a hyperthermophilic archaeon in a dimeric form. *The FEBS Journal* **275**, 2632-2643 (2008).

14. Yeh JI*, et al.* Structural characterizations of glycerol kinase: unraveling phosphorylation-induced long-range activation. *Biochemistry* **48**, 346-356 (2009).

15. Pickl A, Johnsen U, Schönheit P. Fructose degradation in the haloarchaeon *Haloferax volcanii* involves a bacterial type phosphoenolpyruvate-dependent phosphotransferase system, fructose-1-phosphate kinase, and class II fructose-1, 6-bisphosphate aldolase. *J Bacteriol* **194**, 3088-3097 (2012).

16. Sherwood KE, Cano DJ, Maupin-Furlow JA. Glycerol-mediated repression of glucose metabolism and glycerol kinase as the sole route of glycerol catabolism in the haloarchaeon *Haloferax volcanii*. *J Bacteriol* **191**, 4307-4315 (2009).

17. Rawls KS, Martin JH, Maupin-Furlow JA. Activity and transcriptional regulation of bacterial protein-like glycerol-3-phosphate dehydrogenase of the haloarchaea in *Haloferax volcanii*. *J Bacteriol* **193**, 4469-4476 (2011).

18. Hayashi S-I, Lin E. Purification and properties of glycerol kinase from *Escherichia coli*. *Journal of Biological Chemistry* **242**, 1030-1035 (1967).

19. Koga Y, Morikawa M, Haruki M, Nakamura H, Imanaka T, Kanaya S. Thermostable glycerol kinase from a hyperthermophilic archaeon: gene cloning and characterization of the recombinant enzyme. *Protein Engineering, Design and Selection* **11**, 1219-1227 (1998).

20. Atomi H, Fukui T, Kanai T, Morikawa M, Imanaka T. Description of *Thermococcus kodakaraensis* sp. nov., a well studied hyperthermophilic archaeon previously reported as *Pyrococcus* sp. KOD1. *Archaea* **1**, 263 (2004).

21. Lin ECC. Glycerol dissimilation and its regulation in bacteria. *Annual review of microbiology* 535-578 (1976).

22. Cole ST*, et al.* Nucleotide sequence and gene-polypeptide relationships of the glpABC operon encoding the anaerobic sn-glycerol-3-phosphate dehydrogenase of *Escherichia coli* K-12. *J Bacteriol* **170**, 2448-2456 (1988).

23. Varga MER, Weiner JH. Physiological role of GlpB of anaerobic glycerol-3-phosphate dehydrogenase of *Escherichia coli*. *Biochemistry and Cell Biology* **73**, 147-153 (1995).

24. Poblete-Castro I, Wittmann C, Nikel PI. Biochemistry, genetics and biotechnology of glycerol utilization in Pseudomonas species. *Microbial Biotechnology* **13**, 32-53 (2020).

25. Unemoto T, Hayashi M, Hayashi M. Partial purification and properties of respiratory chain-linked L-glycerol 3-phosphate dehydrogenase from a marine bacterium, *Vibrio alginolyticus*. *The Journal of Biochemistry* **90**, 619-628 (1981).

26. Rauchová H, Fato R, Drahota Z, Lenaz G. Steady-state kinetics of reduction of coenzyme Q analogs by glycerol-3-phosphate dehydrogenase in brown adipose tissue mitochondria. *Archives of Biochemistry and Biophysics* **344**, 235-241 (1997).

27. Yeh JI, Chinte U, Du S. Structure of glycerol-3-phosphate dehydrogenase, an essential monotopic membrane enzyme involved in respiration and metabolism. *Proc Natl Acad Sci U S A* **105**, 3280-3285 (2008).

28. Thauer RK, Jungermann K, Decker K. Energy conservation in chemotrophic anaerobic bacteria. *Bacteriological reviews* **41**, 100-180 (1977).

29. Cottingham IR, Ragan CI. Purification and properties of L-3-glycerophosphate dehydrogenase from pig brain mitochondria. *Biochemical Journal* **192**, 9-18 (1980).

30. Hatta T, Inagaki K, Sugio T, Kishimoto N, Tano T. Purification and characterization of glycerol 3-phosphate dehydrogenase from two types of *Acidiphilium* sp. *Agricultural and biological chemistry* **53**, 651-658 (1989).

31. Schryvers A, Weiner JH. The anaerobic sn-glycerol-3-phosphate dehydrogenase of *Escherichia coli*. Purification and characterization. *Journal of Biological Chemistry* **256**, 9959-9965 (1981).

32. Elkhal CK*, et al.* Structure and proposed mechanism of L-α-glycerophosphate oxidase from *Mycoplasma pneumoniae*. *The FEBS Journal* **282**, 3030-3042 (2015).

33. Wijerathne H, Yao H, Wang Y, Lovell S, Battaile KP, Rivera M. Bfd, a new class of [2Fe-2S] protein that functions in bacterial iron homeostasis, requires a structural anion binding site. *Biochemistry* **57**, 5533-5543 (2018).

34. Yao H*, et al.* The structure of the BfrB–Bfd complex reveals protein–protein interactions enabling iron release from bacterioferritin. *Journal of the American Chemical Society* **134**, 13470-13481 (2012).

35. Marchler-Bauer A, Bryant SH. CD-Search: protein domain annotations on the fly. *Nucleic acids research* **32**, W327-W331 (2004).

36. Lin ECC. Glycerol dissimilation and its regulation in bacteria. *Annual Review of Microbiology* **30**, 535-578 (1976).

37. Schryvers A, Lohmeier E, Weiner JH. Chemical and functional properties of the native and reconstituted forms of the membrane-bound, aerobic glycerol-3-phosphate dehydrogenase of *Escherichia coli*. *Journal of Biological Chemistry* **253**, 783-788 (1978).

38. Schryvers A, Weiner JH. The anaerobic sn-glycerol-3-phosphate dehydrogenase of *Escherichia coli*. *The Journal of Biological Chemistry* **256**, 9959-9965 (1981).

39. Austin D, Larson TJ. Nucleotide sequence of the *glpD* gene encoding aerobic sn-glycerol 3-phosphate dehydrogenase of *Escherichia coli* K-12. *J Bacteriol* **173**, 101-107 (1991).

40. Williams TJ, Allen M, Tschitschko B, Cavicchioli R. Glycerol metabolism of haloarchaea. *Environmental microbiology* **19**, 864-877 (2017).

41. de Fátima Alvarez M, Medina R, Pasteris SE, Strasser de Saad AM, Sesma F. Glycerol metabolism of *Lactobacillus rhamnosus* ATCC 7469: cloning and expression of two glycerol kinase genes. *Journal of Molecular Microbiology and Biotechnology* **7**, 170-181 (2004).

42. Tang Y, Shi Y, Jia B, Zhang Y, Wang Q. Evolution and function analysis of glycerol kinase GlpK in *Pseudomonas aeruginosa*. *Biochemical and Biophysical Research Communications* **645**, 30-39 (2023).

43. Králová I, Rigden DJ, Opperdoes FR, Michels PA. Glycerol kinase of *Trypanosoma brucei*: cloning, molecular characterization and mutagenesis. *European journal of biochemistry* **267**, 2323-2333 (2000).

44. Koga Y, Konishi K, Kobayashi A, Kanaya S, Takano K. Anaerobic glycerol-3-phosphate dehydrogenase complex from hyperthermophilic archaeon *Thermococcus kodakarensis* KOD1. *Journal of Bioscience and Bioengineering* **127**, 679-685 (2019).

45. Weiner JH, Heppel LA. Purification of the membrane-bound and pyridine nucleotide-independent L-glycerol 3-phosphate dehydrogenase from *Escherichia coli*. *Biochemical and Biophysical Research Communications* **47**, 1360-1365 (1972).

46. Dawson A, Thorne C. Preparation and some properties of L-3-glycerophosphate dehydrogenase from pig brain mitochondria. *Biochemical Journal* **111**, 27-34 (1969).

47. Wagner M*, et al.* Versatile genetic tool box for the crenarchaeote *Sulfolobus acidocaldarius*. *Front Microbiol* **3**, 214 (2012).

48. Kuschmierz L*, et al.* 5'-untranslated region sequences enhance plasmid-based protein production in *Sulfolobus acidocaldarius Front Microbiol* **15 - 2024**, (2024).

49. Wagner M*, et al.* Investigation of the *malE* promoter and MalR, a positive regulator of the maltose regulon, for an improved expression system in *Sulfolobus acidocaldarius*. *Applied and environmental microbiology* **80**, 1072-1081 (2014).

50. Van der Kolk N, Wagner A, Wagner M, Waßmer B, Siebers B, Albers S-V. Identification of XylR, the activator of arabinose/xylose inducible regulon in *Sulfolobus acidocaldarius*

and its application for homologous protein expression. *Frontiers in Microbiology* **11**, 1066 (2020).
