# Supplementary material for: An unusual glycerol-3-phosphate dehydrogenase in Sulfolobus acidocaldarius elucidates the diversity of glycerol metabolism across Archaea
Source: Commun Biol. 2025 Apr 1;8:539. doi: 10.1038/s42003-025-07953-9 (PMC11962113; doi:10.1038/s42003-025-07953-9)
Supplement: Supplementary file 2 — Description of Additional Supplementary Files [file 42003_2025_7953_MOESM2_ESM.pdf]

Description of Additional Supplementary Files

File Name: Supplementary File 1
Description: LC-MS settings and sample overview.

File Name: Supplementary File 2
Description: Proteome source data for different growth conditions.

File Name: Supplementary File 3
Description: source data for CO-IP experiments.

File Name: Supplementary File 4
Description: Numerical source data for all graphs.
